# Clazosentan Attenuates Endothelin-1-Induced ETA Protein Upregulation and Contractile Sensitization in Brain Pericytes

**DOI:** 10.3390/pharmaceutics18080980

**Published:** 2026-08-09

**Authors:** Genki Chikamatsu, Shinsuke Nakagawa, Yoichi Morofuji, Eri Shiozaki, Yuka Ogawa, Kazuaki Okamura, Yuki Matsunaga, Daisuke Watanabe, Fruzsina R. Walter, Tsuyoshi Izumo, Masami Niwa, Maria A. Deli, Takayuki Matsuo

**Affiliations:** 1 Department of Neurosurgery, Graduate School of Biomedical Sciences, Nagasaki University, Nagasaki 852-8501, Japan; genki3430@gmail.com (G.C.); e.shiozaki15@gmail.com (E.S.); yukaxx0615@gmail.com (Y.O.); ocamoo@yahoo.co.jp (K.O.); y.matsunaga923@gmail.com (Y.M.); takayuki@nagasaki-u.ac.jp (T.M.); 2 Department of Pharmaceutical Care and Health Sciences, Faculty of Pharmaceutical Sciences, Fukuoka University, Fukuoka 814-0180, Japan; shin3@fukuoka-u.ac.jp; 3 Department of Neurosurgery, School of Medicine, Showa Medical University, 1-5-8 Hatanodai, Shinagawa-ku, Tokyo 142-8666, Japan; 4 BBB Laboratory, PharmaCo-Cell Co., Ltd., Nagasaki 852-8135, Japan; watanabe@pharmacocell.co.jp (D.W.); niwa@pharmacocell.co.jp (M.N.); 5 Biological Barriers Research Group, Institute of Biophysics, HUN-REN Biological Research Centre, 6726 Szeged, Hungary; walter.fruzsina@brc.hu (F.R.W.); deli.maria@brc.hu (M.A.D.); 6 Department of Neurosurgery, Gifu University Hospital, Gifu City 501-1194, Japan; izumo.tsuyoshi.n2@f.gifu-u.ac.jp

**Keywords:** clazosentan, endothelin-1, endothelin type A receptor, ETA receptor antagonist, brain pericyte, contractile sensitization, pharmacodynamics, drug–receptor interaction, neurovascular unit

## Abstract

**Background/Objectives**: Brain pericytes are contractile mural cells of the neurovascular unit whose responses to endothelin-1 (ET-1) are mediated primarily by endothelin type A (ETA) receptors. ET-1/ETA signaling has been implicated in pathological pericyte contraction in neurovascular disorders such as Alzheimer’s disease and in cerebrovascular dysfunction after subarachnoid hemorrhage, motivating pharmacological evaluation of selective ETA receptor antagonists at the pericyte level. Clazosentan is a selective ETA receptor antagonist used clinically for cerebral vasospasm; however, its pharmacodynamic effects on brain pericytes remain insufficiently characterized. **Methods**: Pericyte impedance-based contractile and recovery responses to ET-1 were evaluated by xCELLigence real-time cell index analysis. Pericyte viability, morphology, and ETA protein abundance were examined using Cell Counting Kit-8 assay, immunocytochemistry, and Western blotting. As a secondary barrier-related assessment, transendothelial electrical resistance (TEER) was measured in primary rat brain endothelial cell-based in vitro blood–brain barrier models. **Results**: ET-1 induced impedance-based contractile responses with a concentration-related trend, followed by recovery responses, and increased ETA protein abundance in a time- and concentration-related manner; clazosentan significantly attenuated ET-1-induced ETA upregulation. Repeated ET-1 exposure was associated with an enhanced subsequent ET-1-induced impedance-based contractile response and a more sustained response, suggesting contractile sensitization; both effects were significantly attenuated by clazosentan. Clazosentan did not overtly disrupt TEER-assessed barrier properties. **Conclusions**: These findings suggest that ET-1/ETA signaling may shift brain pericytes toward a sensitized contractile response state and that selective ETA blockade by clazosentan attenuates this process without overtly disrupting TEER-assessed barrier properties under the present in vitro conditions. These observations support further pharmacological characterization of clazosentan as a modulator of pericyte ET-1 responses.

## 1. Introduction

Brain pericytes are contractile mural cells of the neurovascular unit that interact closely with endothelial cells on the abluminal surface of capillaries and contribute to vascular stability, microvascular homeostasis, and barrier-related properties [1,2]. Beyond these supporting roles, pericytes are increasingly recognized as active regulators of capillary-level vascular responses through their contractile activity [2,3].

The endothelin system has been implicated in the regulation of pericyte behavior [4]. Endothelin-1 (ET-1) is a potent vasoactive peptide [5] that exerts its contractile effects primarily through endothelin type A (ETA) receptors [6,7]. Within the neurovascular unit, ET-1 released from endothelial cells and other vascular wall-associated cells can act as a paracrine mediator affecting adjacent pericytes [4]. Through its actions on pericytes, ET-1 signaling is considered to contribute to capillary-level vascular responses [3,4,8].

Endothelin-mediated pathological pericyte contraction has been implicated in neurovascular disorders characterized by microcirculatory disturbance, including Alzheimer’s disease and ischemic stroke [9,10]. Endothelin signaling is also strongly associated with cerebral vasospasm and ischemic complications after subarachnoid hemorrhage [11]. These observations suggest that endothelin receptor antagonists may have therapeutic potential in these disorders by modulating endothelin-mediated pericyte contraction. However, the effects of selective ETA blockade on brain pericyte responses and barrier properties related to the blood–brain barrier (BBB) remain insufficiently characterized.

Clazosentan is a potent and selective ETA receptor antagonist developed to inhibit ET-1-mediated vasoconstrictive signaling [12]. Clazosentan is approved in Japan and South Korea for the prevention of cerebral vasospasm after aneurysmal subarachnoid hemorrhage and related ischemic complications [13,14], but has not yet been widely approved in other countries, including the United States. Given its clinical use in the cerebrovascular field, clazosentan is a particularly relevant agent for investigating whether selective ETA blockade can attenuate ET-1-mediated pathological responses in brain pericytes.

In the present study, we used rat brain pericytes and in vitro BBB models to investigate how selective ETA blockade by clazosentan affects ET-1-induced pericyte contractile responsiveness, ETA protein abundance, and transendothelial electrical resistance (TEER)-assessed barrier properties.

## 2. Materials and Methods

### 2.1. Animals

Male Wistar rats aged 3–4 weeks were purchased from Japan SLC, Inc. (Shizuoka, Japan) and used for the isolation of primary rat brain endothelial cells and brain pericytes. All procedures conformed to the National Institutes of Health Guidelines for the Care and Use of Laboratory Animals (NIH Publication No. 80–23) and were approved by the Animal Care and Use Committee of Nagasaki University (Approval No. 2308031889; approval date: 3 August 2023). Animals were euthanized by CO_2_ inhalation, and brain tissues were collected immediately thereafter. No anesthetics or analgesics were administered because no survival procedures were performed, and no agents were administered to live animals in this study. This study is reported in compliance with the ARRIVE guidelines (Animal Research: Reporting In Vivo Experiments).

### 2.2. Materials and Reagents

ET-1 was obtained from Peptide Institute, Inc. (Osaka, Japan; Cat# 4198-v) and dissolved in 0.1% acetic acid to prepare stock solutions. For experimental use, ET-1 was diluted to final concentrations of 0.001, 0.01, 0.1, 1, 10, 100, or 1000 nM, depending on the assay. The final concentration of acetic acid in all experimental conditions was adjusted to 0.001%. ET-1 at 10 nM was selected for subsequent mechanistic experiments as a biologically active, non-maximal concentration within the tested range.

Clazosentan was purchased from MedChemExpress (Monmouth Junction, NJ, USA; Cat# HY-17352) and dissolved in dimethyl sulfoxide (DMSO). The final working concentration was 1 μM. This concentration was chosen to exceed clinically observed total plasma concentrations during continuous infusion (geometric mean 148.2–317.0 ng/mL; ≈0.26–0.55 μM) [15] and to ensure sufficient ETA receptor blockade under in vitro conditions. The DMSO concentration was adjusted to 0.1%.

Corresponding vehicle controls contained 0.001% acetic acid, 0.1% DMSO, or both, depending on the experimental condition.

### 2.3. Cell Culture

Primary rat brain endothelial cells (RBECs) were isolated as previously described [16,17,18,19]. RBECs were maintained in DMEM/F12 supplemented with 10% fetal bovine serum (FBS), basic fibroblast growth factor (1.5 ng/mL), heparin (100 μg/mL), insulin (5 μg/mL), transferrin (5 μg/mL), sodium selenite (5 ng/mL), and gentamicin (50 μg/mL). Cells were maintained at 37 °C in a humidified atmosphere containing 5% CO_2_. Puromycin (4 μg/mL) was added for the first two days to remove contaminating pericytes.

Pericytes were obtained from brain microvessel fragments by culture under selective conditions in uncoated dishes with DMEM containing 10% FBS and antibiotics for two weeks [16]. The medium was replaced every 3 days. Cells were cryopreserved in LABO Banker 2, initially frozen at −80 °C, and then stored in liquid nitrogen until use.

Rat brain pericytes used in the present study were obtained using the same laboratory-established culture system as described in our previous studies [16,17,18,19]. In those studies, the pericyte-enriched nature of this culture system was confirmed by phase-contrast morphology and immunocytochemical staining for pericyte-associated markers, including NG2 and α-smooth muscle actin. No additional immunocytochemical marker verification was performed in the present study. These markers were used to distinguish pericytes from other cell types, such as endothelial, fibroblast-like, or astrocytic cells, rather than to classify pericyte subtypes. Given that these cells enhance endothelial tight-junction barrier properties in BBB co-culture systems, we regarded them as capillary-associated pericytes and used them accordingly in the present study [16,17,18,19]. Pericytes at passage 3 were used for all experiments unless otherwise indicated.

Cultures were routinely inspected by phase-contrast microscopy before seeding onto Transwell inserts or E-Plate 16 plates. Only cultures showing normal morphology and growth characteristics and no visible signs of contamination (e.g., turbidity, debris, or abrupt medium color change) were used. For both TEER and impedance assays, inserts or wells with clearly abnormal morphology or markedly reduced baseline resistance or impedance were predefined for exclusion; no samples met these criteria in the reported datasets.

### 2.4. Real-Time Cell Index Assay

Pericytes were seeded into E-Plate 16 (2.0 × 10^4^ cells/well) and treated with various concentrations of ET-1 on day 5. Impedance was recorded every 10 s for 60 min using the xCELLigence^®^ RTCA system (Agilent Technologies, Inc., Santa Clara, CA, USA) and RTCA Software (Lite 2.2.5). Raw cell index data were exported from RTCA Software and processed in Microsoft Excel to calculate normalized cell index values and relative contraction indices for subsequent statistical analysis.

The xCELLigence system quantifies electrical impedance (cell index), a readout that can reflect multiple processes including changes in cell adhesion, spreading, morphology, and cell–substrate contact area. In this study, following prior work [20], a decrease in cell index was operationally interpreted as an impedance-based contractile response, whereas subsequent recovery of the cell index toward baseline was defined as an impedance-based recovery response and considered to reflect relaxation. Because cell index does not directly measure contractile force and can also be influenced by changes in adhesion and morphology, these terms represent operational interpretations of impedance-based cellular responses rather than direct measurements of contraction or relaxation. All values were normalized to baseline (0 min) and to the mean of the corresponding vehicle control group to control for vehicle effects. The change in cell index was first calculated as (minimum − baseline) / baseline. This value was then normalized to the corresponding vehicle control group to obtain the relative contraction index. Cell index at 60 min was also analyzed.

### 2.5. Cell Viability Assay

Pericytes were seeded into 96-well plates (1.0 × 10^4^ cells/well) on day 0. On day 5, cells were treated with ET-1 at the indicated concentrations. After 48 h, viability was assessed using the Cell Counting Kit-8 (CCK-8; Dojindo Laboratories, Kumamoto, Japan), and absorbance was measured at 450 nm using a TECAN SPARK plate reader. Background absorbance from cell-free wells was subtracted, and values were normalized to the vehicle control group (0.001% acetic acid).

### 2.6. Immunocytochemistry

Pericytes were fixed at 0 (control), 7.5, 15, and 30 min after stimulation with 10 nM ET-1. Cells were fixed in 4% paraformaldehyde for 10 min, permeabilized with 0.1% Triton X-100, and blocked with 3% bovine serum albumin (BSA). F-actin was stained with Acti-Stain™ 555 Phalloidin (Cytoskeleton Inc., Denver, CO, USA; Cat# PHDH1, RRID: not available), reconstituted according to the manufacturer’s instructions and used at a dilution of 1:100, corresponding to a final concentration of 100 nM, for 30 min at room temperature in the dark. Nuclei were counterstained using SlowFade™ Glass Soft-set Antifade Mountant with 4′,6-diamidino-2-phenylindole (DAPI) (Thermo Fisher Scientific, Waltham, MA, USA; Cat# S36920; RRID: not available) according to the manufacturer’s instructions.

ETA immunoreactivity was assessed by immunofluorescence in unstimulated pericytes. Cells were fixed as described above and incubated overnight with rabbit polyclonal anti-endothelin A receptor antibody (Abcam, Cambridge, UK; Cat# ab85163, RRID:AB_2293287) at a dilution of 1:100, followed by fluorescent secondary antibody (Thermo Fisher Scientific, Waltham, MA, USA; Cat# A-11029, RRID:AB_2534088) at a dilution of 1:200 and phalloidin staining. ETA receptor immunoreactivity was visualized as green fluorescence.

Images were acquired using an EVOS^®^ FL Cell Imaging System (Thermo Fisher Scientific, Waltham, MA, USA) equipped with a 20× objective. The same acquisition settings were applied within each staining experiment. Scale bars are indicated in the figure legends. No quantitative image analysis was performed.

### 2.7. Western Blot Analysis

For Western blot analysis, pericytes were treated for 48 h with ET-1 at the indicated concentrations, clazosentan (1 μM), ET-1 plus clazosentan as co-treatment, or the corresponding vehicle controls, unless otherwise indicated. Cells were lysed in radioimmunoprecipitation assay (RIPA) buffer, and protein concentrations were determined using the XL-Bradford [SDS-PAGE applicable] kit (KY-1030; APRO Life Science Institute, Naruto, Tokushima, Japan). A total of 15 μg of protein was loaded per lane, resolved on 12% SDS-PAGE gels (Bio-Rad, Laboratories, Inc., Hercules, CA, USA), transferred to polyvinylidene difluoride (PVDF) membranes, and blocked with Bullet Blocking One (Nacalai Tesque, Inc., Kyoto, Japan). Membranes were probed with rabbit polyclonal anti-endothelin A receptor antibody (Abcam, Cambridge, UK; Cat# ab85163, RRID:AB_2293287) at a dilution of 1:2000 and mouse monoclonal β-actin antibody (Proteintech Group, Inc., Rosemont, IL, USA; Cat# HRP-60008, RRID:AB_2819183) at a dilution of 1:40000. Bands were visualized with Clarity Max enhanced chemiluminescence (ECL) substrate (Bio-Rad Laboratories, Inc., Hercules, CA, USA) and quantified using an iBright FL1500 Imaging System (Thermo Fisher Scientific, Waltham, MA, USA) and iBright Analysis Software version 5.3.0. Total cellular ETA protein signals were normalized to β-actin, and values were expressed relative to the corresponding vehicle control group, which was set to 1.0. For presentation, lanes from the same original blot were cropped and rearranged; non-adjacent lanes are separated by dividing lines. Full uncropped original blot images, including unused lanes and the original lane order, are provided in Supplementary File S1.

### 2.8. Assessment of Impedance-Based Contractile Responsiveness After Repeated ET-1 Exposure

To assess whether repeated ET-1 exposure alters pericyte sensitivity to subsequent ET-1 stimulation, pericytes were pretreated with ET-1, ET-1 plus clazosentan, or the corresponding vehicle for 48 h. The 48 h pretreatment period was selected to align the functional sensitization experiment with the exposure period used to assess sustained changes in ETA protein abundance. After pretreatment, cells were washed, detached, reseeded into E-Plate 16 in drug-free medium, and stimulated with 10 nM ET-1 or the corresponding vehicle. Relative contraction indices were calculated from baseline and minimum values, and cell index at 60 min was also analyzed. In this study, “contractile sensitization” was used as an operational term to describe the enhanced impedance-based contractile response to ET-1 stimulation after prior ET-1 exposure, without implying a specific underlying signaling mechanism.

In a separate washout/reseeding control experiment, pericytes were pretreated with clazosentan (1 μM) or DMSO for 48 h, washed, detached, reseeded in drug-free medium, and stimulated with ET-1 (10 nM) or the corresponding vehicle to assess whether residual clazosentan exposure affected the subsequent acute ET-1 response.

### 2.9. Exploratory Assessment of the Effect of Short-Term Clazosentan Exposure on the ET-1-Induced Impedance-Based Contractile Response

Pericytes were pretreated with clazosentan (1 μM) or DMSO on day 4. On day 5, 10 nM ET-1 or the corresponding vehicle was added without washing out clazosentan or DMSO, and impedance was monitored for 60 min. Cell index values were normalized to the corresponding vehicle control at each time point, and relative contraction indices were calculated from baseline and minimum values.

### 2.10. In Vitro Blood–Brain Barrier Models

Two models were established: an endothelial monoculture (EOO) and an endothelial–pericyte co-culture (EPO). cellQART^®^ inserts (SABEU GmbH & Co. KG, Northeim, Germany) were coated with collagen IV and fibronectin at 100 μg/mL for the endothelial side and with collagen IV at 100 μg/mL for the pericyte side. RBECs (1.0 × 10^5^ cells/insert) were seeded onto the upper surface of collagen IV- and fibronectin-coated inserts (0.4 μm pore size; 24-well format). In co-culture conditions, pericytes (1.0 × 10^4^ cells/insert) were seeded onto the underside of collagen IV-coated membranes and allowed to adhere overnight. RBECs were seeded on the following day. Both systems were maintained in RBEC medium supplemented with 500 nM hydrocortisone.

### 2.11. Transendothelial Electrical Resistance (TEER)

TEER measurements were performed using a CellZscope device with CellZscope software version 4.4.8 (nanoAnalytics GmbH, Münster, Germany). Values from cell-free inserts were subtracted from those with cells, and results were expressed in Ω·cm^2^. TEER was continuously recorded, and values at 0, 24, and 48 h post-treatment were used for analysis.

### 2.12. Drug Treatment in the BBB Model

Following baseline TEER measurement (0 h), clazosentan (1 μM), ET-1 (10 nM), or the corresponding vehicle was applied to the luminal side of the inserts. The same concentrations used in the pericyte experiments were applied to the BBB models to assess whether these experimental treatment conditions overtly affected TEER-assessed barrier properties. For normalized analyses, TEER values at each time point were first expressed relative to the corresponding vehicle control group at the same time point and then corrected using the 0 h value of the same insert as baseline.

### 2.13. Statistical Analysis

Sample size is reported as *n*, defined as the number of technical replicates, corresponding to individual parallel wells or inserts analyzed under the same experimental condition. When applicable, values were normalized to the corresponding within-assay vehicle control before group comparisons.

Experiments were not conducted in a blinded fashion. However, TEER and impedance data were acquired automatically by the instrument, and outcomes were quantified using pre-specified, objective metrics applied uniformly across groups.

Data are presented as mean ± SD for datasets judged to be normally distributed and as median [IQR] for non-normally distributed datasets, where IQR indicates the interquartile range, defined as the 25th–75th percentile range. For datasets with *n* ≤ 8, normality was considered unreliable; therefore, single-time-point datasets are presented as median [IQR] and were analyzed using nonparametric methods regardless of normality test results. In contrast, longitudinal TEER datasets were analyzed using a repeated-measures framework to account for within-sample correlation over time.

A priori quality-control criteria were applied to identify technically invalid measurements. Measurements showing patterns clearly attributable to technical failure, such as recording or handling artifacts that were incompatible with the assay principle, were treated as technical artifacts and excluded from analysis. After exclusion of technical artifacts, statistical outliers were assessed within each experimental group using the Tukey IQR rule (*k* = 1.5) and adjusted by Winsorization to the nearest non-outlying value before statistical testing.

Normality was assessed using the Shapiro–Wilk test. Homogeneity of variance was assessed using the Brown–Forsythe and Bartlett’s tests, where applicable. For comparisons among three or more groups, datasets with normal distribution and equal variances were analyzed using ordinary one-way ANOVA followed by Tukey’s multiple-comparisons test. Datasets with normal distribution but unequal variances were analyzed using the Brown–Forsythe and Welch one-way ANOVA followed by Dunnett’s T3 multiple-comparisons test. Non-normally distributed datasets were analyzed using the Kruskal–Wallis test followed by Dunn’s multiple comparisons test. For nonparametric comparisons between two independent groups, the Mann–Whitney U test was used. Longitudinal TEER data were analyzed using two-way repeated-measures ANOVA with each insert treated as a repeated-measures unit and with Geisser–Greenhouse correction, followed by Šídák’s multiple-comparisons test for between-group comparisons at each time point, when appropriate. Spearman’s rank correlation coefficient was used for concentration–response and association analyses, using the mean value for each concentration as a single observation.

All statistical analyses were performed using GraphPad Prism (v10.6.1; GraphPad Software, Boston, MA, USA). A two-sided *p* value < 0.05 was considered statistically significant.

### 2.14. Use of Artificial Intelligence Tools

ChatGPT (GPT-5, OpenAI, San Francisco, CA, USA) was used under author supervision to assist with Python (v3.14.3; Python Software Foundation, Wilmington, DE, USA) scripting for data visualization and figure formatting guidance. The tool was not used to generate or modify raw data, perform final statistical analyses, or draw scientific conclusions. All statistical analyses and final results were generated and verified by the authors using GraphPad Prism (v10.6.1). The authors reviewed the relevant outputs and take full responsibility for the content of this manuscript.

## 3. Results

### 3.1. ET-1 Induces Impedance-Based Contractile Responses in Pericytes with a Concentration-Related Trend

To investigate ET-1-induced pericyte responses, we performed real-time impedance analysis using the xCELLigence^®^ system, in which a decrease in cell index was operationally interpreted as an impedance-based contractile response. Pericytes were stimulated with increasing concentrations of ET-1, and cell index was recorded every 10 s for 60 min.

**Figure 1:**
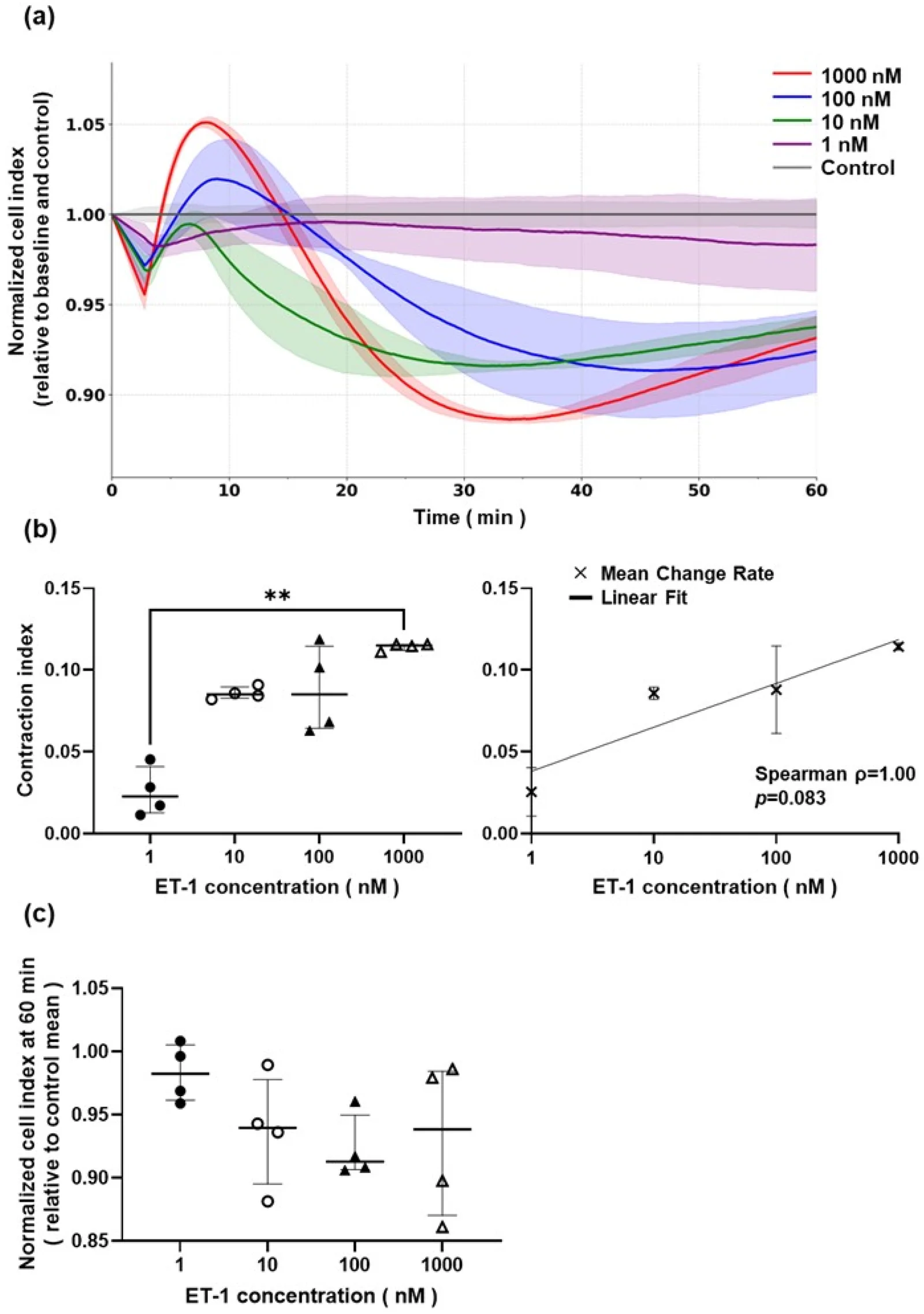
ET-1 induces an impedance-based contractile response in pericytes with a concentration-related trend measured by xCELLigence real-time impedance analysis. (**a**) Time course of normalized cell index over 60 min after stimulation with ET-1 (1, 10, 100, or 1000 nM). For visualization of the time course, data are shown as mean ± SD (*n* = 4). (**b**) Relative contraction indices calculated for each ET-1 concentration and compared across groups. (**c**) Normalized cell index at 60 min after ET-1 stimulation. Data in (**b**,**c**) are presented as median [IQR] (*n* = 4). Statistical analysis was performed using the Kruskal–Wallis test with Dunn’s multiple comparisons test. Spearman’s rank correlation was used to assess the association between ET-1 concentration and impedance-based contractile response magnitude. ** *p* < 0.01. Abbreviations: ET-1, endothelin-1; nM, nanomolar.

As shown in Figure 1a, ET-1 evoked a multiphasic time-course profile consisting of an initial transient contractile phase, a transient recovery phase, a predominant sustained contractile phase, and a later recovery phase. The magnitude of this response differed across ET-1 concentrations. To quantify the response, relative contraction indices were calculated for each ET-1 concentration and compared across groups. This analysis demonstrated a greater impedance-based contractile response in the 1000 nM ET-1 group than in the 1 nM ET-1 group (*p* = 0.0065; Figure 1b). In addition, although the correlation did not reach statistical significance, Spearman’s rank analysis showed a strong positive monotonic association between ET-1 concentration and impedance-based contractile response magnitude (Spearman’s ρ = 1.00, *p* = 0.083; Figure 1b), supporting a concentration-related trend toward increasing impedance-based contractile response magnitude at higher ET-1 concentrations. To quantify the recovery phase, we compared the normalized cell index at 60 min across ET-1 concentrations. No significant differences were detected among groups at this time point (Figure 1c).

These findings indicate that ET-1 induces impedance-based contractile responses in pericytes, with a concentration-related trend toward greater response magnitude at higher ET-1 concentrations.

### 3.2. ET-1 Does Not Affect Pericyte Viability Across a Wide Range of Concentrations

To determine whether ET-1 exerts cytotoxic effects on pericytes, cell viability was assessed after 48 h treatment with a range of ET-1 concentrations (0.001–1000 nM) using a colorimetric assay.

**Figure 2:**
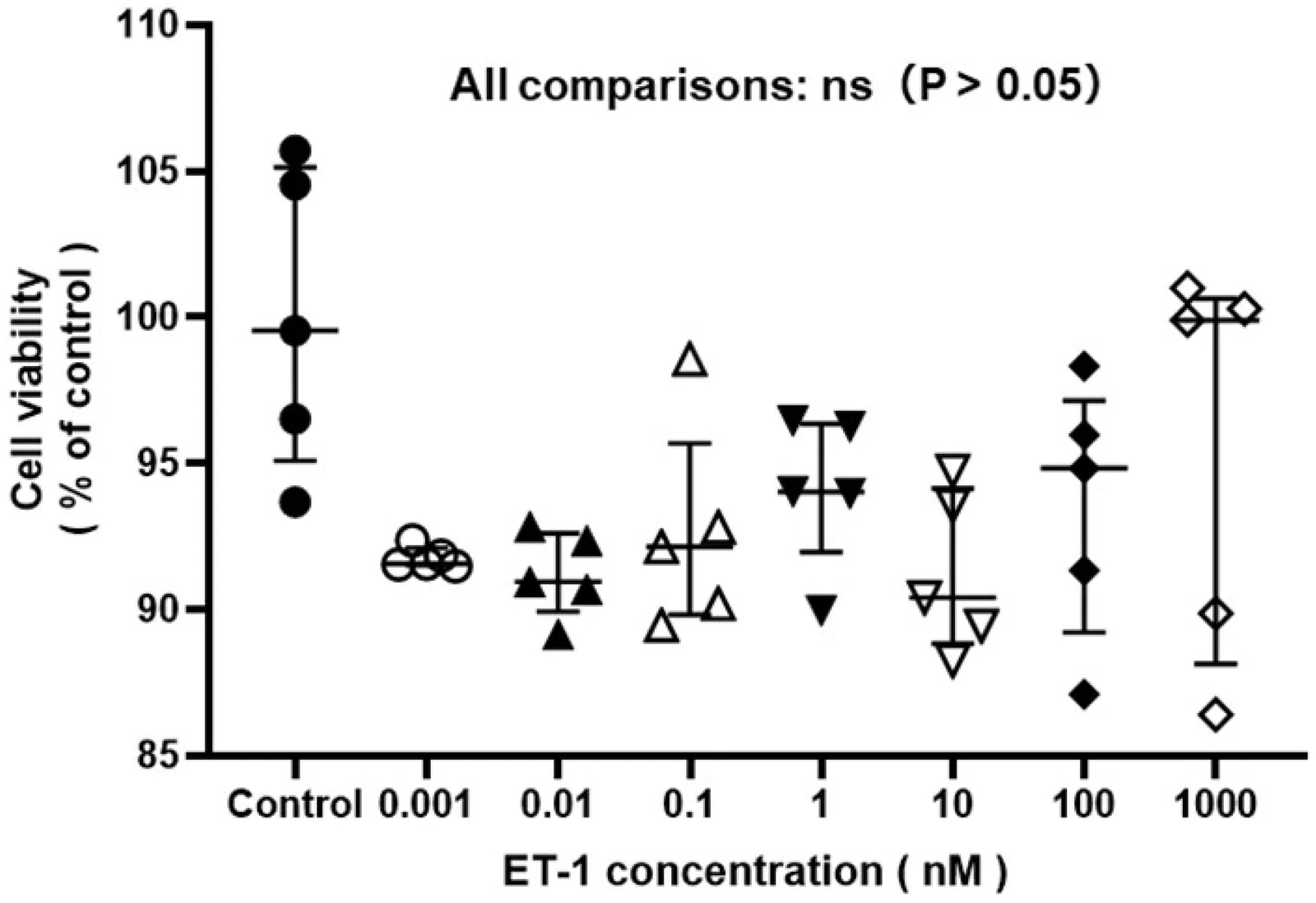
ET-1 does not affect pericyte viability across a wide range of concentrations. Pericytes were treated with ET-1 (0.001–1000 nM) for 48 h. Cell viability (CCK-8) is expressed as % of vehicle control (0.001% acetic acid). Data are presented as median [IQR] (*n* = 5 wells per group). Statistical analysis was performed using the Kruskal–Wallis test with Dunn’s multiple comparisons test; all comparisons were not significant (*p* > 0.05). Abbreviations: ET-1, endothelin-1; nM, nanomolar.

As shown in Figure 2, ET-1 did not significantly alter pericyte viability at any tested concentration. Viability values remained close to control levels across the concentration range, and no clear dose-dependent trend was observed. Statistical analysis using the Kruskal–Wallis test with Dunn’s multiple comparisons test revealed no significant differences among groups (*p* > 0.05 for all comparisons).

These findings indicate that ET-1 is not cytotoxic to pericytes across the tested concentration range, supporting the interpretation that the observed impedance-based contractile responses are not secondary to reduced cell viability.

### 3.3. ET-1 Induces Morphological Changes Suggestive of Contraction in Pericytes, and ETA Immunoreactivity Is Detectable Under Baseline Conditions

**Figure 3:**
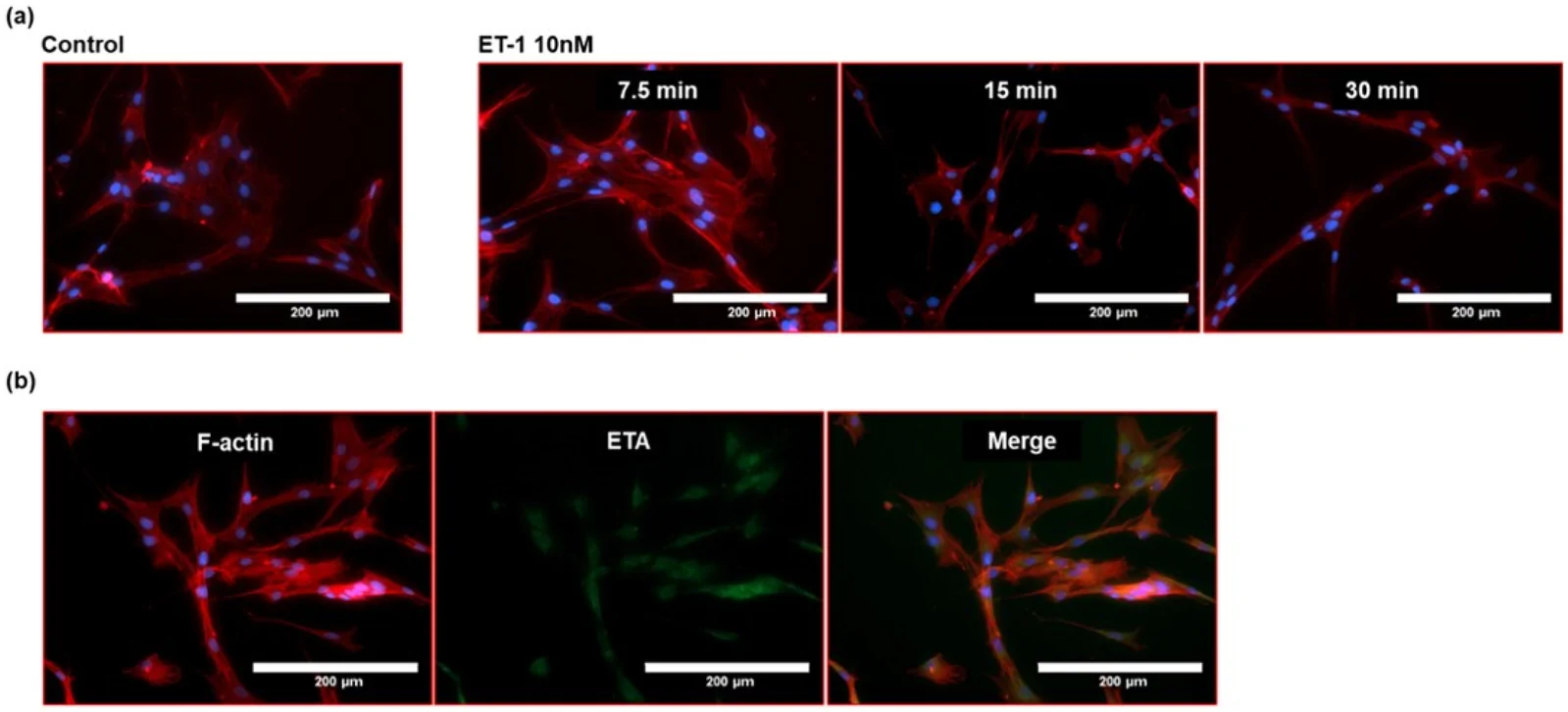
ET-1 induces morphological changes suggestive of contraction in pericytes, and ETA immunoreactivity is detectable under baseline conditions. (**a**) F-actin (red) and nuclei (blue) in pericytes treated with ET-1 (10 nM) for 7.5, 15, and 30 min. Control field is shown for comparison. ET-1 induced time-dependent morphological changes suggestive of contraction. (**b**) ETA immunoreactivity (green) in unstimulated pericytes by immunofluorescence. F-actin: Acti-Stain™ 555 phalloidin (red); nuclei: DAPI (blue). Images are representative; no quantitative image analysis was performed. Scale bar = 200 μm. Abbreviations: ET-1, endothelin-1; ETA, endothelin type A receptor.

To investigate the acute effects of ET-1 on pericyte morphology, we performed time-course immunofluorescence imaging following ET-1 stimulation (10 nM). As shown in Figure 3a, ET-1 induced time-dependent morphological changes suggestive of contraction in rat brain pericytes. Compared to the untreated control, ET-1-treated cells exhibited progressive thinning and elongation of both cell bodies and processes, with the most prominent changes observed at 30 min post-stimulation. These morphological alterations were visualized using F-actin staining and were assessed qualitatively in representative fields. No quantitative image analysis was performed.

To determine the basal expression of ETA receptors in pericytes, we performed immunostaining under unstimulated conditions. ETA immunoreactivity was clearly observed in pericytes in representative fields (Figure 3b), indicating constitutive receptor expression. ETA staining was observed throughout the cells, but subcellular localization could not be definitively assessed. These findings show that ET-1 induces qualitative morphological changes suggestive of contraction and that ETA immunoreactivity is detectable in unstimulated pericytes.

### 3.4. ET-1 Increases ETA Receptor Protein Abundance in a Time- and Concentration-Related Manner, Which Is Attenuated by Clazosentan

**Figure 4:**
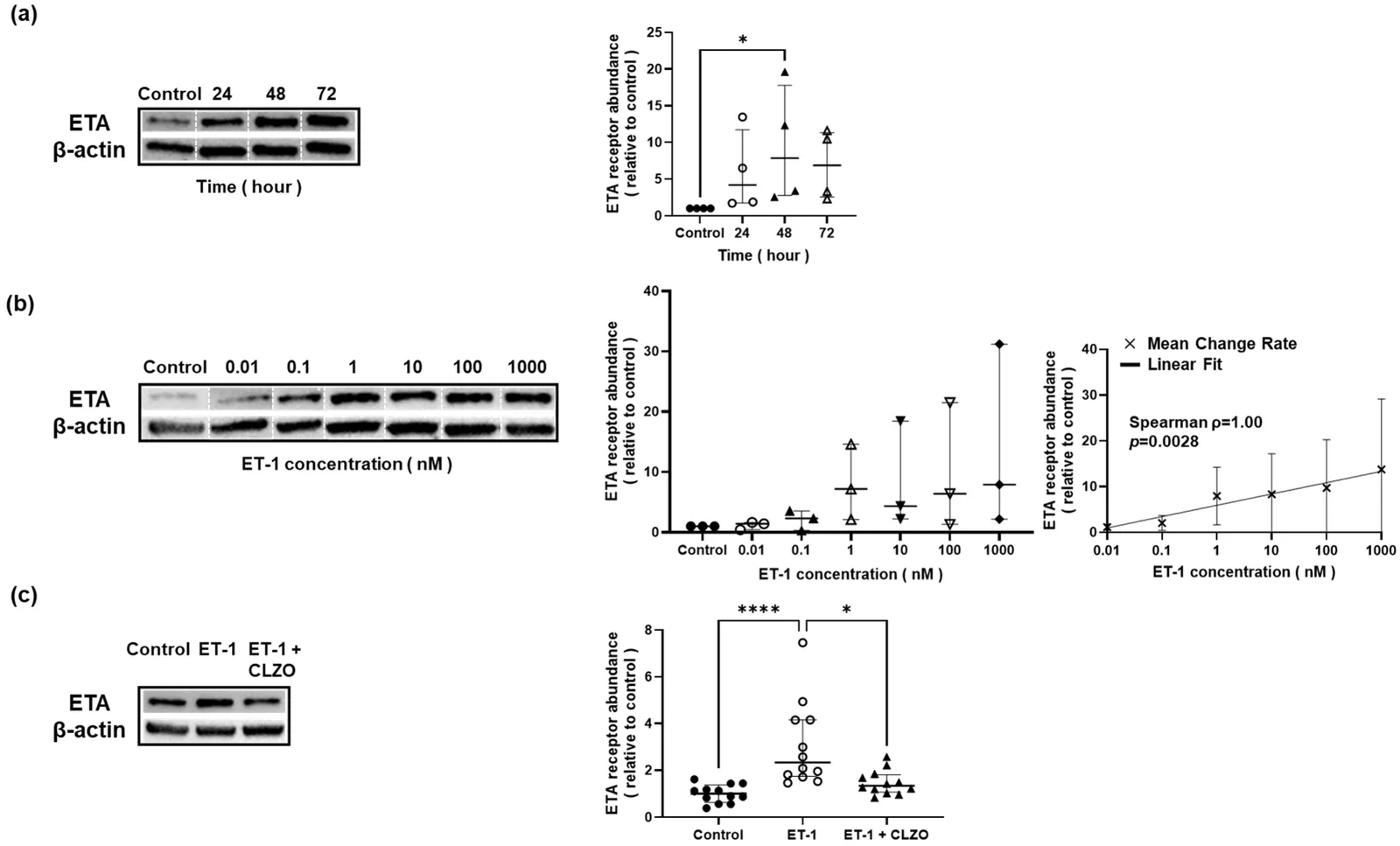
Clazosentan attenuates ET-1-induced ETA protein upregulation. Representative Western blots and quantification of ETA protein normalized to β-actin and expressed relative to control are shown. (**a**) Time course of ETA protein abundance after ET-1 (10 nM) stimulation for 24, 48, and 72 h (median [IQR], *n* = 4). (**b**) Concentration–response of ETA protein abundance after ET-1 (0.01–1000 nM) stimulation for 48 h (median [IQR], *n* = 3 per concentration). Spearman’s rank correlation was used to assess the association between ET-1 concentration and ETA protein abundance. (**c**) Effect of clazosentan (CLZO; 1 μM) on ET-1 (10 nM, 48 h)-induced ETA upregulation (median [IQR], *n* = 12 per group). Statistical analysis was performed using the Kruskal–Wallis test with Dunn’s multiple comparisons test. * *p* < 0.05, **** *p* < 0.0001. Representative cropped blots are shown. Selected lanes from the same original blot were cropped and arranged for presentation, and non-adjacent lanes are separated by dividing lines. Full uncropped blot images, including the original lane order, unused lanes, molecular weight markers, and bands used for quantification, are provided in Supplementary File S1; original TIFF files are provided as Supplementary File S2. Abbreviations: ET-1, endothelin-1; ETA, endothelin type A receptor; CLZO, clazosentan.

To examine whether ET-1 alters the abundance of its receptor ETA in pericytes, we performed Western blot analysis under time-course, concentration–response, and clazosentan co-treatment conditions. Representative blot images and corresponding quantification are shown in Figure 4.

In the time-course experiment (Figure 4a), ET-1 increased ETA receptor protein abundance over time, reaching statistical significance at 48 h compared with the control group (*p* = 0.0268), whereas no significant differences were observed at 24 or 72 h (*p* = 0.2603 and *p* = 0.1223, respectively).

In the concentration–response experiment (Figure 4b), pericytes were treated with increasing concentrations of ET-1 (0.01–1000 nM) for 48 h. ETA protein abundance increased with ET-1 concentration, and Spearman’s rank correlation demonstrated a strong positive monotonic association between ET-1 concentration and ETA protein abundance (ρ = 1.00, *p* = 0.0028). Although post hoc pairwise comparisons did not identify statistically significant differences between individual concentration groups after correction, the overall monotonic relationship supports a concentration-related increase in ETA protein abundance.

Co-treatment with the selective ETA receptor antagonist clazosentan (1 μM) significantly attenuated ET-1-induced ETA protein upregulation (Figure 4c). ET-1 markedly increased ETA protein abundance compared with control (*p* < 0.0001), while the addition of clazosentan significantly reduced this effect (ET-1 vs. ET-1 + clazosentan, *p* = 0.0238). No significant difference was observed between the control and ET-1 + clazosentan groups (*p* = 0.1970), indicating suppression of ET-1-mediated ETA upregulation.

These findings indicate that ET-1 enhances ETA protein abundance in pericytes in a time- and concentration-related manner and that this effect can be attenuated by ETA receptor blockade.

### 3.5. ET-1 Pretreatment Is Associated with ETA Protein Upregulation and an Enhanced Subsequent ET-1-Induced Impedance-Based Contractile Response, Which Is Attenuated by Clazosentan

To determine whether ET-1 pretreatment conditions associated with ETA protein upregulation alter pericyte sensitivity to subsequent ET-1 stimulation, pericytes were pretreated for 48 h with ET-1, ET-1 plus clazosentan, or vehicle, then reseeded and re-stimulated with ET-1 in the xCELLigence assay. Impedance-based contractile responses were quantified by contraction amplitude (relative contraction index) and the normalized cell index at 60 min.

**Figure 5:**
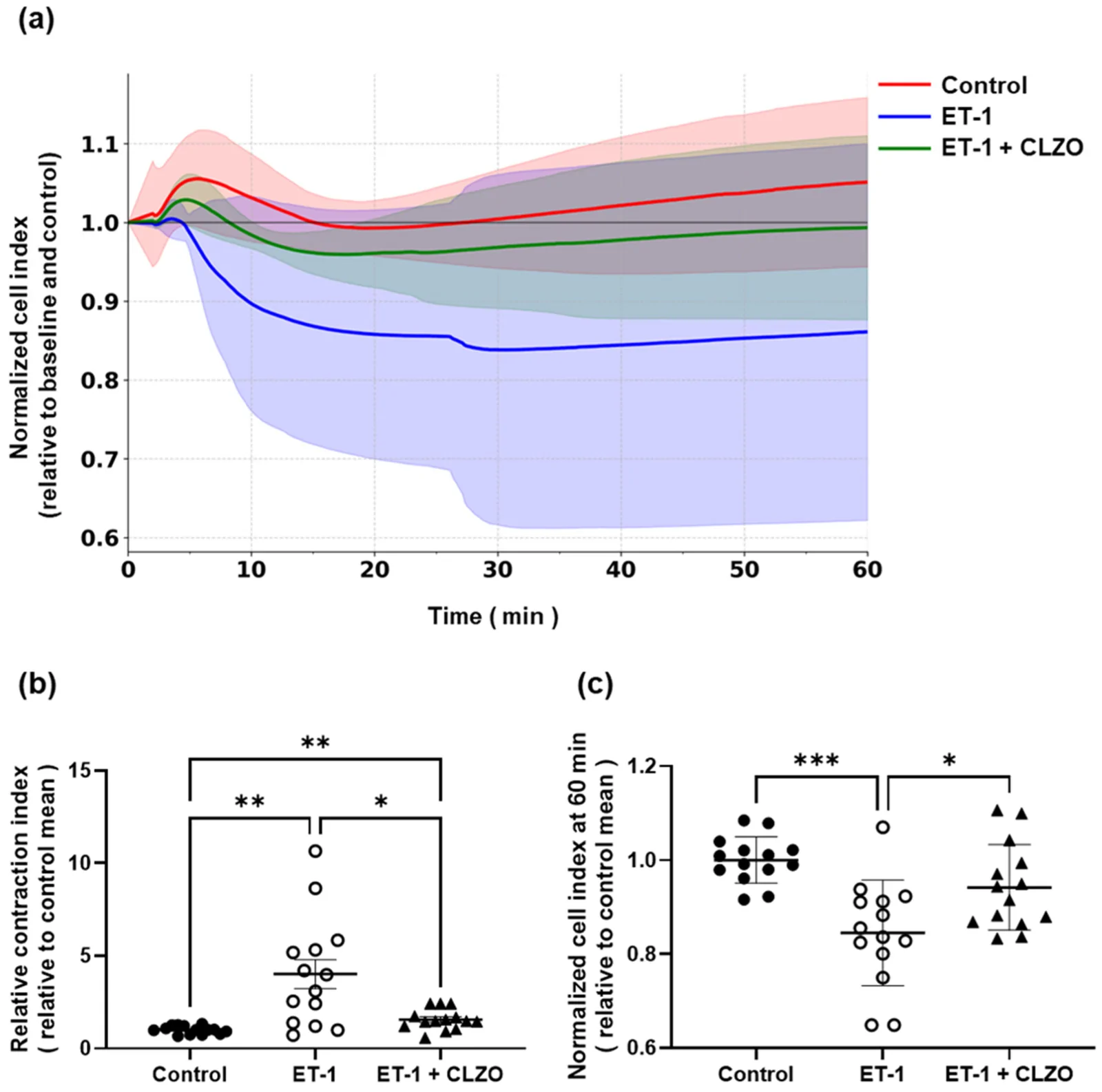
ET-1 pretreatment is associated with an enhanced subsequent ET-1-induced impedance-based contractile response, which is attenuated by clazosentan. Pericytes were pretreated for 48 h with ET-1 (10 nM), ET-1 + clazosentan (CLZO; 1 μM), or vehicle, then reseeded and stimulated with ET-1 (10 nM). (**a**) Time course of normalized cell index over 60 min. (**b**) Contraction amplitude expressed as the relative contraction index, normalized to the control mean. (**c**) Normalized cell index at 60 min (relative to the control mean). Data are presented as mean ± SD (*n* = 14 per group). Statistical analysis: (**b**) Brown–Forsythe test and Welch one-way ANOVA with Dunnett’s T3 multiple comparisons test; (**c**) ordinary one-way ANOVA with Tukey’s multiple comparisons test. * *p* < 0.05, ** *p* < 0.01, *** *p* < 0.001. Abbreviations: ET-1, endothelin-1; CLZO, clazosentan.

As shown in Figure 5a, ET-1-pretreated pericytes exhibited a greater decrease in normalized cell index over 60 min compared with vehicle control, while this effect was attenuated by co-treatment with clazosentan.

In Figure 5b, ET-1 pretreatment significantly enhanced contraction amplitude compared with control (*p* = 0.0062). The ET-1 + clazosentan group remained significantly greater than control (*p* = 0.0082), and contraction amplitude was significantly lower in the ET-1 + clazosentan group than in the ET-1 group (*p* = 0.0241), indicating partial attenuation by clazosentan.

In Figure 5c, the normalized cell index at 60 min was significantly reduced in the ET-1 group compared with control (*p* = 0.0001). This sustained response was attenuated by clazosentan, such that the ET-1 + clazosentan group was not significantly different from control (*p* = 0.1994) but remained significantly different from the ET-1 group (*p* = 0.0163).

These results indicate that ET-1 pretreatment was associated with an enhanced subsequent ET-1-induced impedance-based contractile response, suggesting contractile sensitization in association with ETA protein upregulation. Clazosentan attenuated this enhanced contractile responsiveness.

A washout/reseeding control experiment indicated no detectable residual effect of clazosentan on acute ET-1-evoked impedance-based contractile response (Figure S1). In addition, clazosentan pretreatment resulted in a numerically smaller acute ET-1-evoked impedance-based contractile response during short-term stimulation, but this difference did not reach statistical significance (Figure S2; Mann–Whitney U test, *p* = 0.2000). Given the limited sample size, this supplementary analysis was considered exploratory and was not interpreted as definitive evidence of acute clazosentan-mediated inhibition.

Full statistical results are provided in Supplementary Table S1.

### 3.6. Clazosentan Does Not Overtly Disrupt TEER-Assessed Barrier Properties in the In Vitro BBB Models

As a secondary barrier-related assessment, we examined whether the concentrations of clazosentan and ET-1 used in the pericyte experiments affected TEER-assessed barrier properties in two in vitro BBB models: brain endothelial monoculture (EOO) and brain endothelial–pericyte co-culture (EPO). TEER measurements were conducted at 0, 24, and 48 h following luminal administration of clazosentan (1 μM), ET-1 (10 nM), or the corresponding vehicle. The 48 h time point was selected to match the prolonged exposure period used in the pericyte experiments.

**Figure 6:**
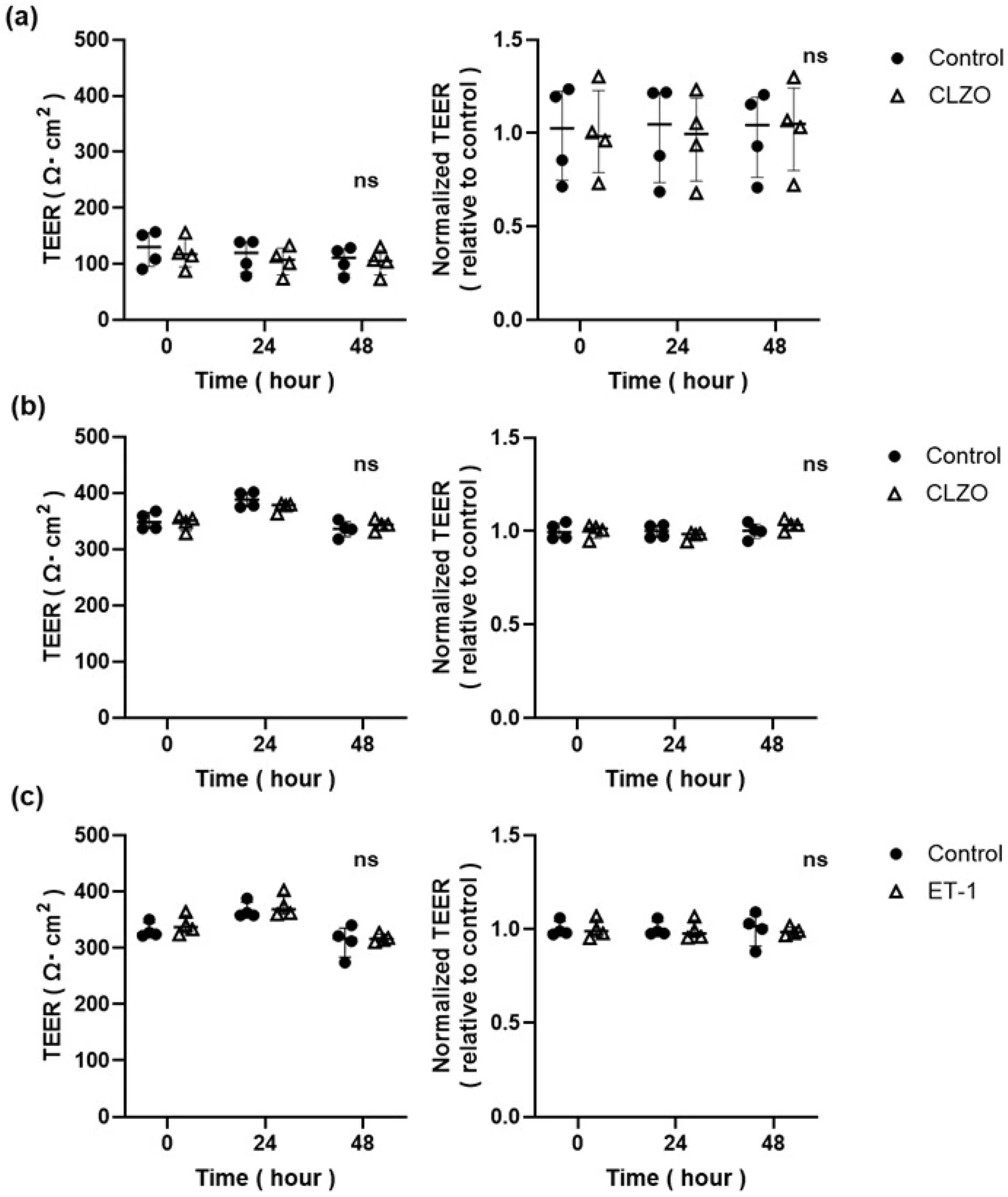
Effects of clazosentan and ET-1 on TEER in BBB models. (**a**) TEER in endothelial monoculture, vehicle vs. CLZO. (**b**,**c**) TEER in endothelial–pericyte co-culture ((**b**), vehicle vs. CLZO; (**c**), vehicle vs. ET-1). CLZO was applied at 1 μM and ET-1 at 10 nM, and TEER was evaluated at 0, 24, and 48 h after treatment. Left panels: absolute TEER (Ω·cm^2^); right panels: normalized TEER (relative to control). Data are presented as median [IQR] (*n* = 4 inserts per group). Longitudinal TEER data were analyzed using two-way repeated-measures ANOVA followed by Šídák’s multiple comparisons test; ns, not significant. Abbreviations: TEER, transendothelial electrical resistance; ET-1, endothelin-1; CLZO, clazosentan; BBB, blood–brain barrier.

In the EOO model, clazosentan treatment did not induce any significant change in TEER over time compared with the vehicle group (Figure 6a). Similarly, in the EPO model, neither clazosentan nor ET-1 significantly altered TEER values (Figure 6b,c). Notably, as established in previous studies [16,17], baseline TEER values were consistently higher in EPO than in EOO, reflecting the contribution of pericytes to barrier function.

TEER data are presented as absolute values (Ω·cm^2^) and as normalized values (Figure 6a–c), and are expressed as median [IQR] (*n* = 4 inserts per group). Longitudinal TEER data were analyzed using two-way repeated-measures ANOVA followed by Šídák’s multiple comparisons test, with each insert treated as a repeated-measures unit. Šídák-adjusted multiple comparisons showed no significant between-group differences at any time point. Full statistical results, including main effects, interaction terms, and adjusted post hoc comparisons, are provided in Supplementary Table S1. These results indicate that clazosentan, at the concentration tested, did not overtly disrupt TEER-assessed barrier properties in either BBB model under the present experimental conditions.

**Figure 7:**
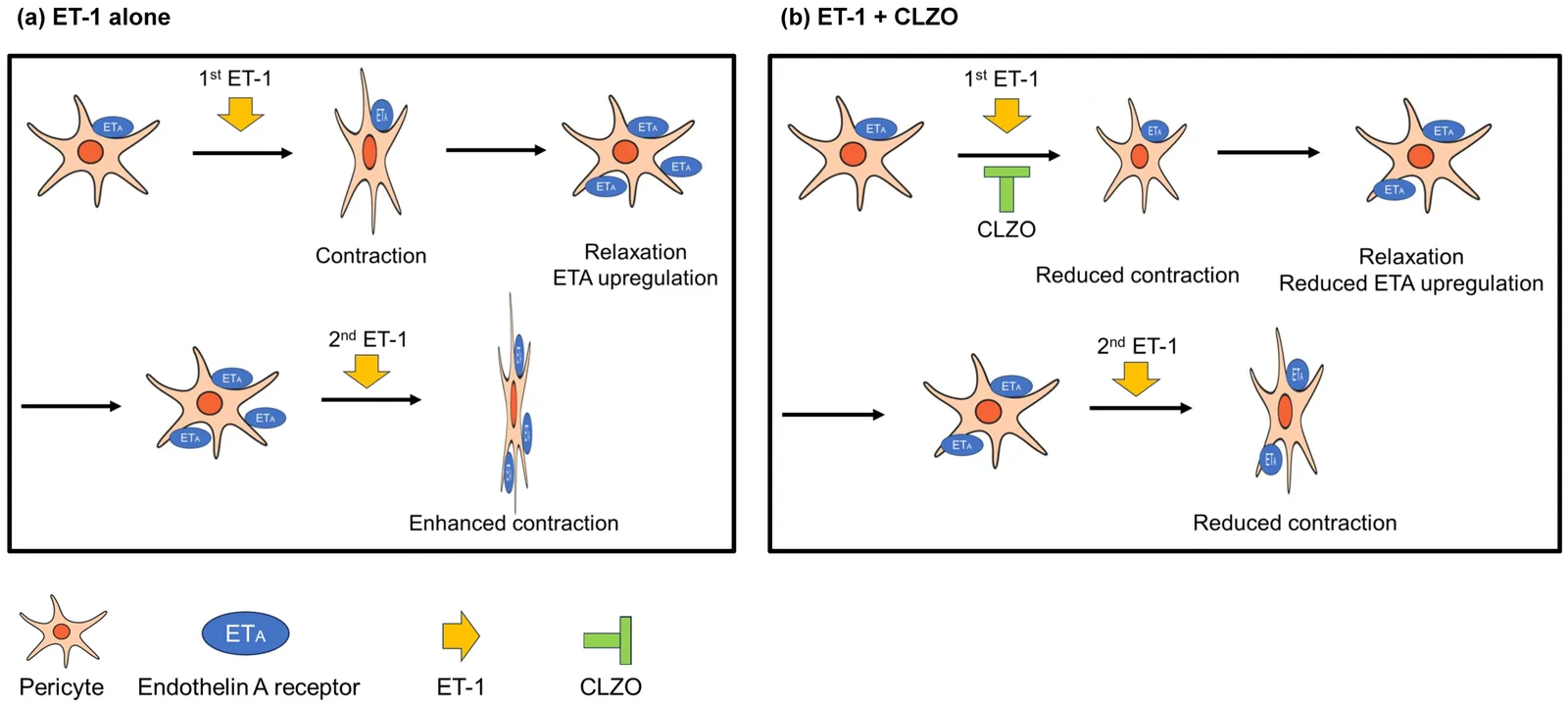
Schematic illustration of clazosentan’s effects on ET-1-induced ETA protein upregulation and contractile sensitization in brain pericytes. (**a**) Without clazosentan, ET-1 induces a contractile response in pericytes and increases ETA protein abundance; repeated ET-1 exposure is associated with enhanced subsequent contractile response. (**b**) With clazosentan, ET-1-induced ETA protein upregulation and the associated enhancement of subsequent contractile response are attenuated. This schematic summarizes the attenuating effects of clazosentan on ET-1-induced ETA protein upregulation and contractile sensitization. Abbreviations: ET-1, endothelin-1; ETA, endothelin type A receptor; CLZO, clazosentan.

The principal findings regarding the effects of clazosentan on ET-1-induced ETA protein upregulation and contractile sensitization in brain pericytes are summarized schematically in Figure 7.

## 4. Discussion

In the present study, repeated ET-1 exposure in brain pericytes was associated with increased ETA protein abundance and enhanced responsiveness to subsequent ET-1 stimulation, and both changes were attenuated by clazosentan. In parallel, clazosentan did not significantly alter TEER in the in vitro BBB models. Together, these findings suggest that selective ETA blockade by clazosentan may modulate ET-1-related changes in pericyte response state without overtly disrupting TEER-assessed barrier properties under the present experimental conditions. A schematic summary of these findings is presented in Figure 7.

ET-1 induced impedance-based contractile responses in pericytes, an interpretation supported by the qualitative morphological changes suggestive of contraction observed in the present study. This is consistent with earlier work showing that brain pericytes respond to ET-1 [4] and with electrical-impedance studies demonstrating ET-1-evoked contractile responses in cultured human brain-derived pericytes [8]. Previous work further indicates that increasing ET-1 concentration augments the magnitude, and to some extent the duration, of the response [8]. Consistent with this, the principal effect of acute ET-1 exposure in Figure 1 appears to be an increase in the overall excursion of the response within a fixed observation window, rather than a marked prolongation of the impedance-based recovery response. Moreover, because cell viability was preserved after ET-1 exposure, the observed decrease in impedance is unlikely to reflect cytotoxicity or cell loss, further supporting its interpretation as an impedance-based contractile response. The relatively slow time course observed here also aligns better with prior descriptions of capillary pericyte behavior than with the rapid phasic responses of arteriolar smooth muscle cells [21,22,23].

A central finding of the present study is that pericyte responsiveness to ET-1 was not static. Basal ETA immunoreactivity was detectable in cultured pericytes, ET-1 exposure increased ETA protein abundance in a time- and concentration-related manner, and clazosentan attenuated this increase. Moreover, in repeated ET-1 exposure experiments, conditions associated with greater ETA protein abundance showed enhanced responses to subsequent ET-1 stimulation, and this enhancement was likewise attenuated by clazosentan. Together, these observations support a possible functional link between ETA protein upregulation and the sensitized response state associated with repeated ET-1 exposure. However, the present experiments do not directly establish causality, and this mechanistic link should therefore be regarded as an inference based on the observed association.

This interpretation is supported by the broader vascular literature. Across several vascular and contractile-cell contexts, increased ETA expression or ETA-dependent signaling has been associated with enhanced ET-1 responsiveness, augmented contractile phenotype, or calcium sensitization [24,25,26,27,28,29]. In this respect, the ET-1-induced increase in ETA observed in the present pericyte model is biologically plausible, although comparable evidence in brain pericytes remains more limited than in vascular smooth muscle. This point should nevertheless be interpreted cautiously, because the increase in ETA identified here was based primarily on antibody-based detection of total cellular ETA protein abundance and does not distinguish functional surface receptors from intracellular receptor pools. The present data therefore do not establish that repeated ET-1 exposure increased functional surface ETA per se. Nevertheless, because the rise in ETA protein abundance was accompanied by enhanced subsequent ET-1 responsiveness and was attenuated by clazosentan, it is unlikely to represent biologically irrelevant protein accumulation alone. At minimum, the data support the possibility that repeated ET-1 exposure alters the ET-1 response state of brain pericytes in a manner that favors enhanced contractile sensitization.

Comparison of the waveform profiles after single and repeated ET-1 exposure provides additional insight into the nature of the sensitized response observed in Figure 5. For descriptive clarity, the ET-1-evoked impedance waveform may be considered to comprise four phases: an initial transient contractile phase, a transient recovery phase, a predominant sustained contractile phase, and a later recovery phase. No canonical four-phase nomenclature has been established for ET-1-induced pericyte responses, and this framework should therefore be regarded as a descriptive synthesis informed by the brain pericyte impedance literature, retinal pericyte Ca^2+^ studies, and the broader vascular smooth muscle literature [8,28,30].

Within this descriptive framework, the early phase may reflect ETA-coupled Gq/phospholipase C/inositol 1,4,5-trisphosphate signaling and intracellular Ca^2+^ mobilization, whereas the later sustained phase may involve sustained Ca^2+^ entry and calcium sensitization, including store-operated calcium entry/Orai-, TMEM16A-, and RhoA/ROCK–myosin light-chain phosphatase-related mechanisms reported in pericyte or smooth muscle studies [22,23,28,29]. The transient recovery phase may correspond to the interval during which the initial Ca^2+^ rise subsides before these sustained mechanisms predominate, and the later recovery phase may reflect progressive reversal of those processes. These mechanistic assignments were not directly examined in the present study and should therefore be regarded as mechanistically informed inferences rather than demonstrated mechanisms.

This waveform comparison further suggests that the sensitized response in Figure 5 cannot be explained solely by the observed increase in ETA protein abundance. In the single-exposure experiment, conditions showing greater contractile responses still exhibited a relatively distinct early transient recovery. After ET-1 pretreatment, by contrast, the condition with the strongest subsequent response no longer showed a comparably evident early recovery. ET-1 pretreatment therefore appeared not only to be associated with increased ETA protein abundance and enhanced ET-1 responsiveness, but also to increase the likelihood that the response would progress into, and remain within, a stronger sustained contractile state. This pattern raises the possibility that, in addition to enhanced contractile responsiveness associated with increased ETA protein abundance, prior ET-1 exposure altered the cellular state such that recovery after the contractile response became less efficient. Consistent with this interpretation, ET-1 has been linked to phenotypic change in brain pericytes under diabetes-mimicking conditions [31], while in other contractile-cell systems ET-1/ETA signaling has been associated with sustained contractile phenotypes and calcium sensitization [29], as well as stress fiber formation and focal adhesion maturation [27]. In this context, clazosentan may also help limit the transition toward an exaggerated and prolonged contractile response with impaired recovery, thereby helping to maintain a more stable pericyte response state.

These observations may be relevant to neurovascular disorders in which ET-1 signaling is increased within the neurovascular unit. In Alzheimer’s disease, ET-1 is elevated, and experimental evidence supports the involvement of amyloid-β (Aβ) in capillary constriction mediated by pericyte ETA signaling, which may contribute to hypoperfusion and downstream neurovascular consequences [9,32]. Elevated ET-1 has also been reported in the cerebrospinal fluid of patients with ischemic stroke and aneurysmal subarachnoid hemorrhage [33,34]. Although the present study does not model these disorders directly, such contexts raise the possibility that repeated or sustained endothelin stress could contribute to pericyte dysfunction, microcirculatory instability, and broader neurovascular unit disturbance [3,9,31]. Within that framework, our data offer a possible cellular basis by which ET-1 exposure may shift the pericyte response state toward greater subsequent responsiveness and a more persistent contractile response. The present findings suggest that, in addition to inhibiting ETA-mediated vasoconstrictive signaling in larger cerebral vessels, clazosentan may exert pharmacodynamic effects at the microvascular cellular level by attenuating ET-1-associated contractile responses and the enhanced responsiveness observed after repeated ET-1 exposure in brain pericytes. Such pericyte-level modulation may complement the established vascular actions of clazosentan, although the present in vitro findings do not directly demonstrate improved cerebral perfusion, prevention of delayed ischemic complications, or clinical benefit.

An additional pharmacological consideration is the receptor-subtype selectivity of clazosentan. Fluid retention and pulmonary complications, including pulmonary edema and pleural effusion, have been reported during clazosentan treatment [15,35]. Experimental studies with other ETA-selective antagonists suggest that selective ETA blockade may enhance signaling through unblocked endothelin type B (ETB) receptors, thereby increasing vascular permeability and promoting fluid retention [36]; however, this mechanism has not been directly demonstrated for clazosentan. In the injured brain, ETB receptors are upregulated in reactive astrocytes, and ETB receptor blockade has been reported to attenuate BBB disruption and brain edema in experimental models [37]. Thus, although speculative, an altered balance between ETA- and ETB-mediated signaling during selective ETA blockade could potentially influence BBB permeability and edema-related responses. Because the present study did not assess ETB receptor signaling, include astrocytes in the BBB model, or directly evaluate tracer permeability, this possibility remains unresolved and warrants further investigation.

In the BBB experiments, clazosentan did not significantly alter TEER in either endothelial monoculture or endothelial–pericyte co-culture, and ET-1 alone likewise did not produce a measurable TEER change. These results indicate that, in the static in vitro BBB model used here, clazosentan did not overtly compromise barrier function as assessed by TEER. The ET-1 result, however, should be interpreted more cautiously, because previous studies suggest that the effects of ET-1 on the BBB are not uniform and may depend on interactions with astrocytes and inflammatory stimuli [38], while ET-1 has also been implicated in barrier-related endothelial changes under pathological conditions [39,40]. Accordingly, the absence of a TEER change in the present study should not be taken to mean that ET-1 is irrelevant to BBB dysfunction, but rather that, under the conditions used here, its effect was not detectable by this readout. Thus, although the present TEER data suggest no overt adverse effect of clazosentan on TEER-assessed barrier properties, TEER alone is insufficient to fully characterize its effects on BBB function or brain pericytes. Further evaluation using additional readouts will be required to define the effects of clazosentan.

Several limitations should be acknowledged. First, this study was performed in simplified static in vitro systems using cultured rat brain pericytes and BBB models; therefore, the findings should be interpreted as controlled cellular pharmacological responses rather than as direct evidence of clazosentan efficacy in complex neurovascular environments. In vivo neurovascular pathology involves multiple interacting processes, including inflammatory signaling, oxidative stress, endothelial dysfunction, and fibrotic or phenotypic remodeling, none of which were directly assessed in the present study and each of which may influence pericyte contractile responses, endothelin receptor regulation, and BBB function. Second, pericyte identity in the present experiments was based on the previously established characterization of this culture system, and no additional marker-based verification was performed in the current study; some degree of culture heterogeneity or phenotypic variation therefore cannot be excluded. Third, some experiments were based on relatively small numbers of technical replicates rather than independent biological replicates, limiting assessment of biological variability. Fourth, the 1 μM clazosentan concentration used in vitro does not necessarily reflect the pharmacologically active exposure achieved in vivo; because clazosentan is highly bound to plasma proteins and the free-drug concentration was not determined under our culture conditions, direct comparison with unbound plasma concentrations or actual drug exposure in the brain perivascular compartment should be interpreted with caution. Fifth, the impedance-based contractile response used in this study is an operational measure of contraction-associated changes in cell behavior and does not directly quantify contractile force, as changes in cell shape, adhesion, spreading, and related cell–substrate interactions may also contribute to the cell index signal; no orthogonal assay directly quantifying contractile force, such as traction force microscopy or collagen gel contraction, was performed. Sixth, BBB-related effects were evaluated exclusively by TEER, which primarily reflects electrical resistance to ionic passage and tight-junction-related barrier properties, whereas permeability to soluble tracers, transcytosis, inflammatory responses, and more complex multicellular neurovascular interactions were not assessed. Finally, because Western blotting quantified total cellular ETA protein abundance, the present study does not determine whether the observed increase reflects altered transcription, protein turnover, trafficking, or receptor stabilization, nor whether it corresponds to increased cell-surface ETA receptor abundance. The observed association between ETA upregulation and enhanced subsequent ET-1 responsiveness does not establish causality, and downstream signaling mechanisms such as calcium dynamics, RhoA/Rho-associated protein kinase signaling, myosin light-chain kinase signaling, and extracellular signal-regulated kinase signaling were not directly examined. These limitations indicate that the present findings define an in vitro pharmacological response pattern in which clazosentan modulates ET-1/ETA-dependent pericyte sensitization, warranting further validation in more complex BBB and neurovascular models.

## 5. Conclusions

Taken together, this study demonstrates that repeated ET-1 exposure in brain pericytes is associated with increased ETA protein abundance and enhanced responsiveness to subsequent ET-1 stimulation, whereas clazosentan attenuates both ETA upregulation and contractile sensitization without overtly disrupting TEER-assessed barrier properties in the in vitro BBB models used here. These findings identify ET-1/ETA-related pericyte sensitization as a pharmacologically modifiable cellular response and suggest that clazosentan may contribute to the modulation of pericyte responsiveness under conditions of repeated or sustained endothelin stress.

## Data Availability

The data supporting the conclusions of this study are included within the article and Supplementary Materials. Additional datasets are available from the corresponding author upon reasonable request.

## Supplementary Materials

The following supporting information can be downloaded at: https://www.mdpi.com/article/10.3390/pharmaceutics18080980/s1, Figure S1: Washout/reseeding control experiment assessing residual effects of clazosentan on acute ET-1-evoked impedance-based contractile response; Figure S2: Exploratory short-term clazosentan pretreatment experiment; Table S1: Full statistical results; File S1: Annotated full uncropped Western blot images with the original lane order; File S2: Original uncropped Western blot image files in TIFF format.

## Abbreviations

The following abbreviations are used in this manuscript:

Aβ	Amyloid β
BBB	Blood–brain barrier
BSA	Bovine serum albumin
CCK-8	Cell Counting Kit-8
CLZO	Clazosentan
DAPI	4′,6-diamidino-2-phenylindole
DMEM/F12	Dulbecco’s modified Eagle medium/nutrient mixture F-12
DMSO	Dimethyl sulfoxide
ECL	Enhanced chemiluminescence
EOO	Endothelial monoculture
EPO	Endothelial–pericyte co-culture
ET-1	Endothelin-1
ETA	Endothelin type A receptor
ETB	Endothelin type B receptor
FBS	Fetal bovine serum
IQR	Interquartile range
PVDF	Polyvinylidene difluoride
RBECs	Rat brain endothelial cells
RhoA/ROCK	Ras homolog family member A/Rho-associated protein kinase
RIPA	Radioimmunoprecipitation assay
RTCA	Real-time cell analysis
TEER	Transendothelial electrical resistance
