# Supplementary material for: Clazosentan Attenuates Endothelin-1-Induced ETA Protein Upregulation and Contractile Sensitization in Brain Pericytes
Source: Pharmaceutics. 2026 Aug 9;18(8):980. doi: 10.3390/pharmaceutics18080980 (PMC13516358; doi:10.3390/pharmaceutics18080980)
Supplement: Supplementary file 1 [file pharmaceutics-18-00980-s001.zip › Supplementary Figures S1 and S2.pdf]

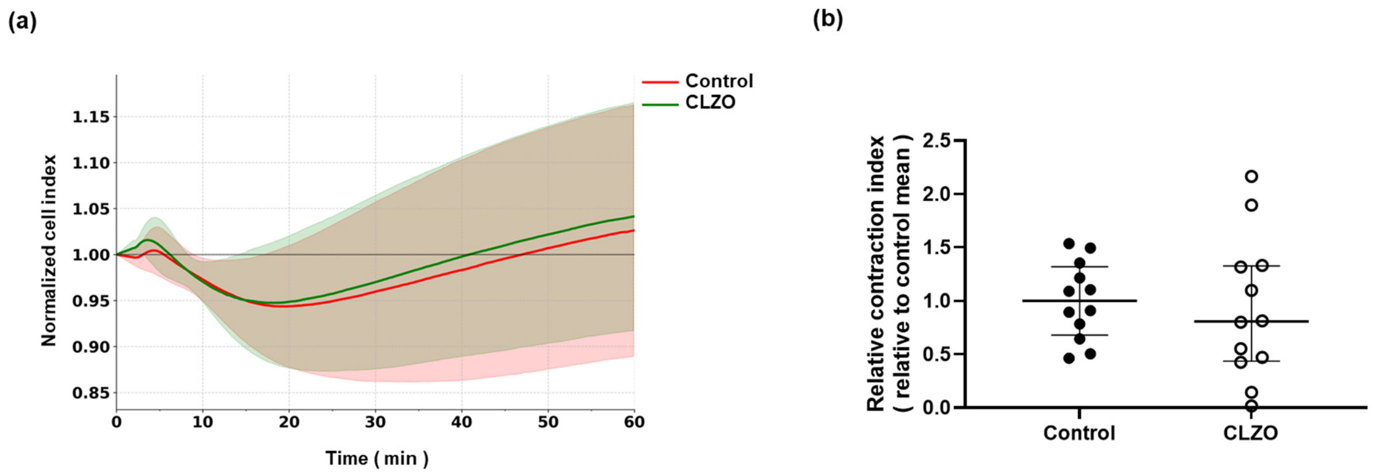

Supplementary Figure S1. Washout/reseeding control for potential clazosentan carryover.

Pericytes were pretreated with clazosentan or vehicle for 48 h, then harvested, washed, and reseeded prior to uniform ET-1 stimulation.

(a) Time course of normalized cell index following ET-1 stimulation. For visualization of the time course, data are shown as mean ± SD ($n = 12$).

(b) Contraction amplitude expressed as the relative contraction index. Data are presented as median [IQR] ($n = 12$ per group). No significant difference in contraction amplitude was observed between groups (unpaired t test, $p > 0.05$; 95% CI of the mean difference −0.5342 to 0.3747). Variability was also not significantly different between groups (F test, $p = 0.0557$).

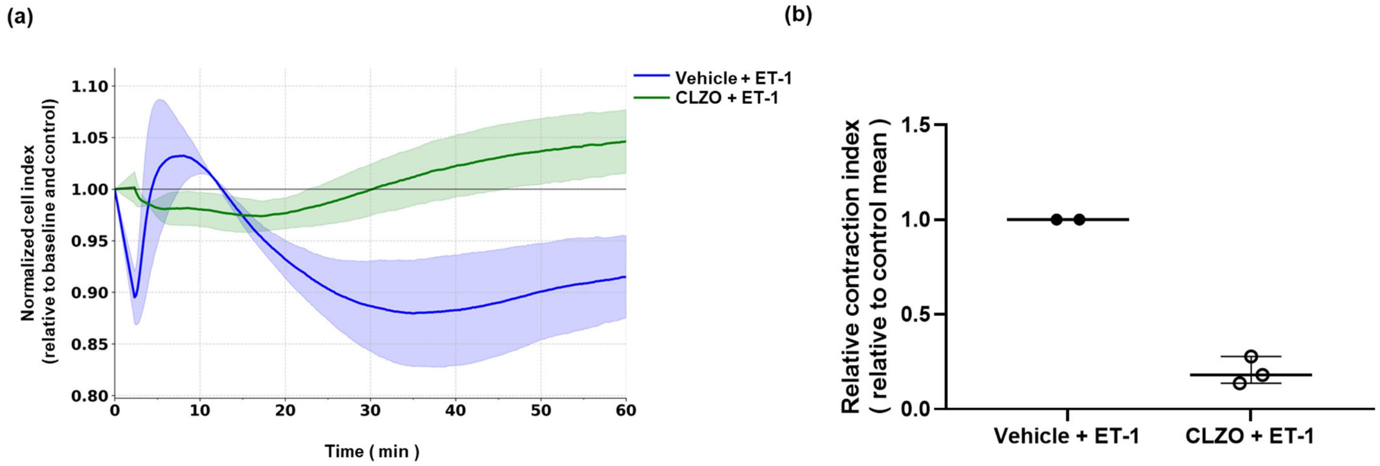

Supplementary Figure S2. Assessment of acute ETA blockade on ET-1–evoked impedance-based contractile response.

Pericytes were pretreated with clazosentan (1 µM) or vehicle (0.1% DMSO) for 24 h, followed by ET-1 stimulation (10 nM).

(a) Time course of normalized cell index during short-term stimulation. For visualization of the time course, data are shown as mean ± SD (vehicle n = 2; CLZO n = 3).

(b) Contraction amplitude was numerically lower in the clazosentan group, but this difference was not statistically significant. Data are presented as median [IQR] (vehicle n = 2; CLZO n = 3). Statistical analysis was performed using the Mann–Whitney U test; p = 0.2000.

Abbreviations: ET-1, endothelin-1; DMSO, dimethyl sulfoxide; CLZO, clazosentan.
