# Supplementary material for: Clazosentan Attenuates Endothelin-1-Induced ETA Protein Upregulation and Contractile Sensitization in Brain Pericytes
Source: Pharmaceutics. 2026 Aug 9;18(8):980. doi: 10.3390/pharmaceutics18080980 (PMC13516358; doi:10.3390/pharmaceutics18080980)
Supplement: Supplementary file 1 [file pharmaceutics-18-00980-s001.zip › Supplementary File S1_Annotated_Full_Uncropped_Western_Blots.pdf]

Supplementary File S1. Full unedited blot images

Original, uncropped blot images corresponding to the cropped blots shown in Figure 4.Red boxes indicate the lanes included in the cropped blots shown in the main figure. Arrows indicate the bands used for quantification. Molecular weight marker positions are indicated in kDa.

Full unedited blot for Figure 4a (ETA)

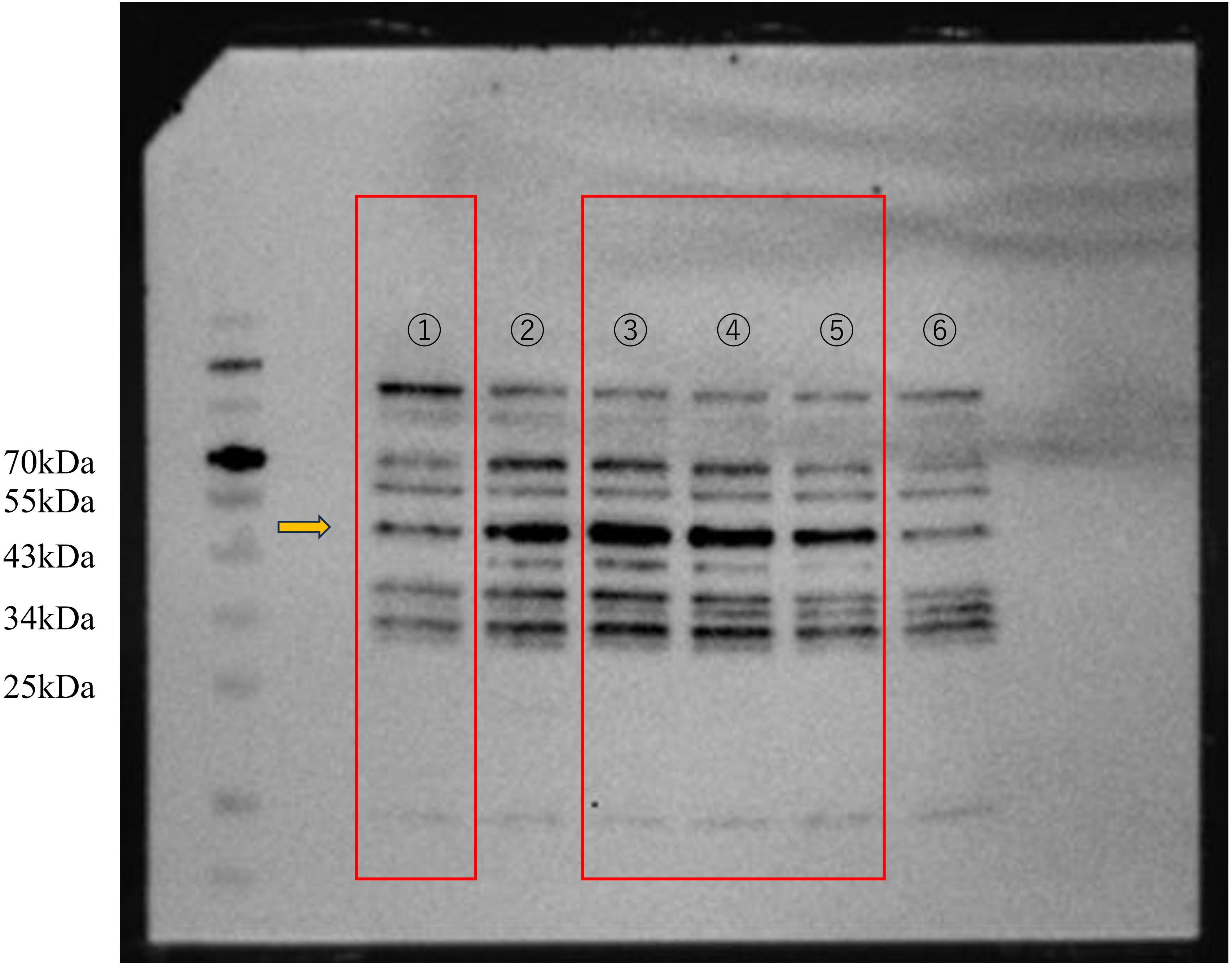

① Control (Acetic acid)
② Not used (ET-1 96hr)
③ ET-1 72hr
④ ET-1 48hr
⑤ ET-1 24hr
⑥ Not used (ET-1 45min)

Arrow indicates the band used for quantification.

Full unedited blot for Figure 4a (β-actin)

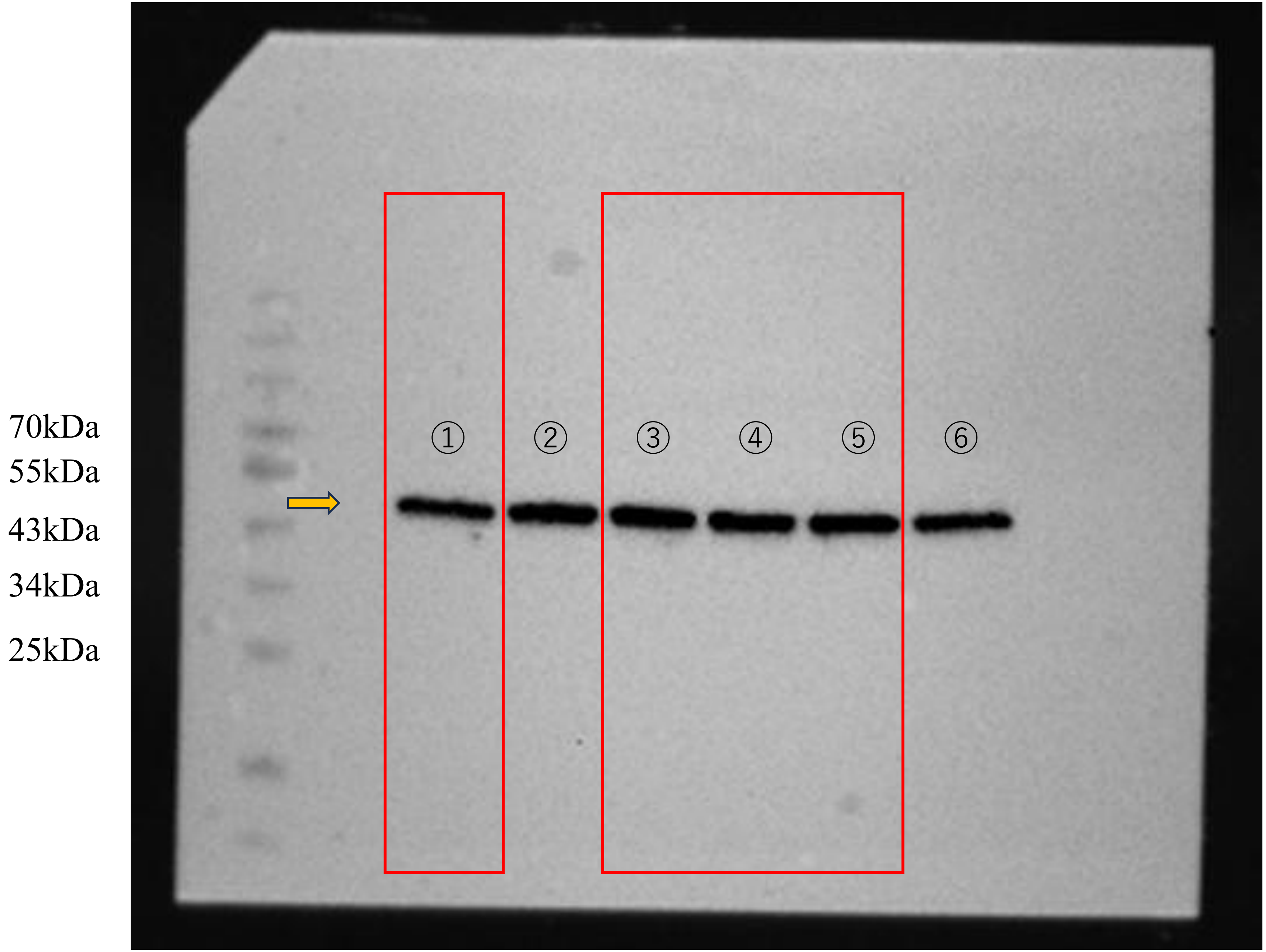

① Control (Acetic acid)
② Not used (ET-1 96hr)
③ ET-1 72hr
④ ET-1 48hr
⑤ ET-1 24hr
⑥ Not used (ET-1 45min)

Arrow indicates the band used for quantification.

Full unedited blot for Figure 4b (ETA)

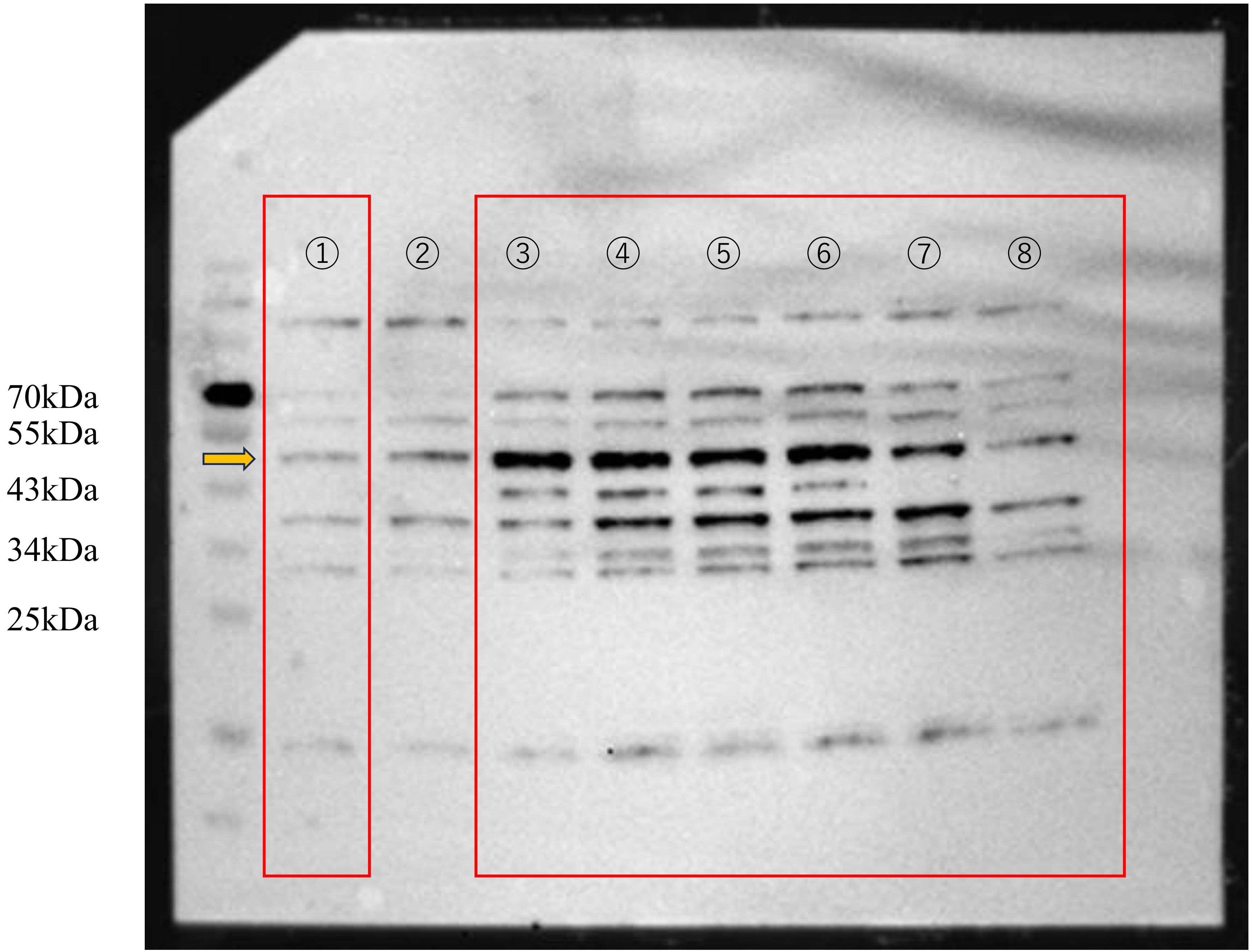

① Control (Acetic acid)
② Not used (Acetic acid + DMSO)
③ ET-1 1000 nM
④ ET-1 100 nM
⑤ ET-1 10 nM
⑥ ET-1 1 nM
⑦ ET-1 0.1 nM
⑧ ET-1 0.01 nM

Arrow indicates the band used for quantification.

Full unedited blot for Figure 4b (β-actin)

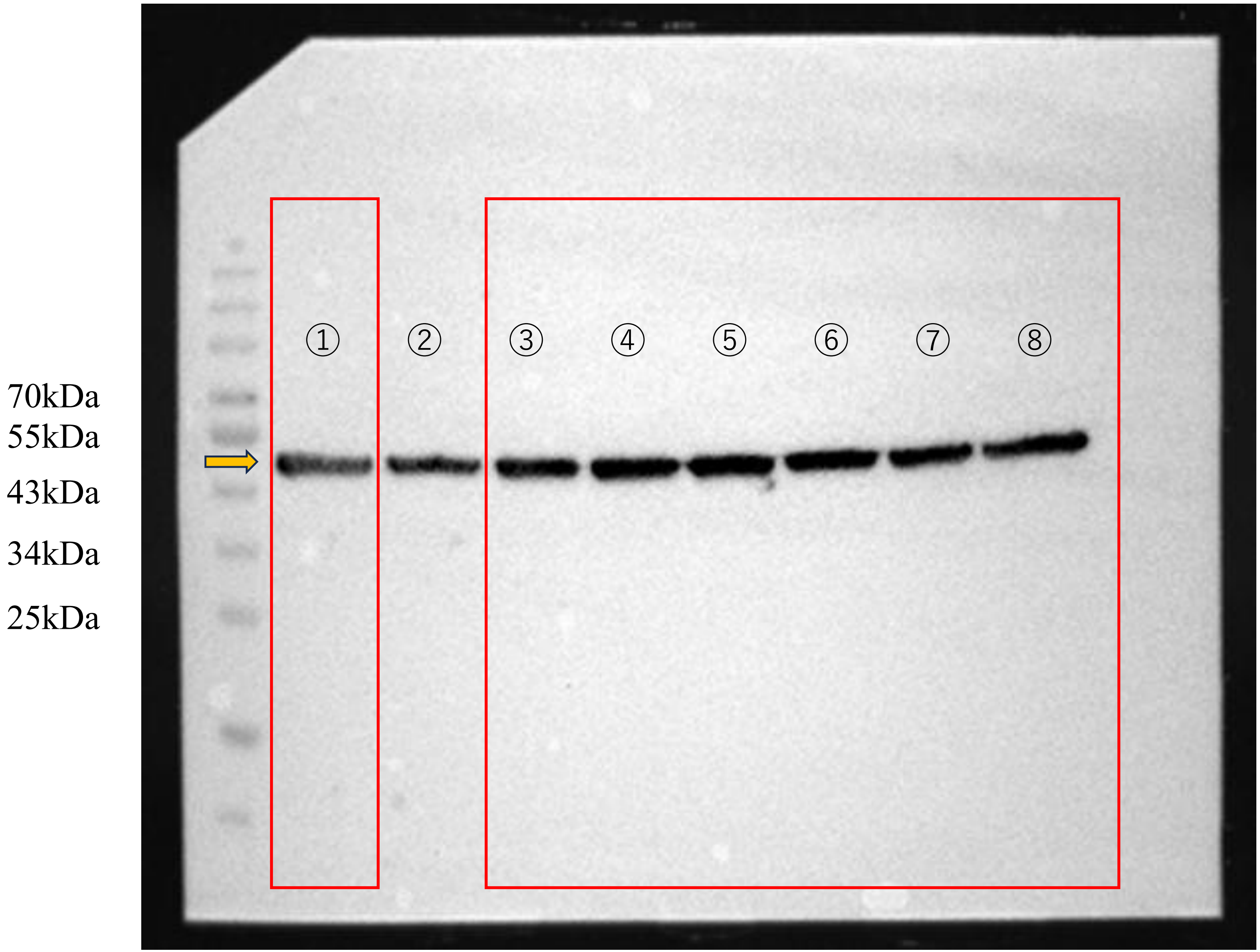

① Control (Acetic acid)
② Not used (Acetic acid + DMSO)
③ ET-1 1000 nM
④ ET-1 100 nM
⑤ ET-1 10 nM
⑥ ET-1 1 nM
⑦ ET-1 0.1 nM
⑧ ET-1 0.01 nM

Arrow indicates the band used for quantification.

Full unedited blot for Figure 4c (ETA)

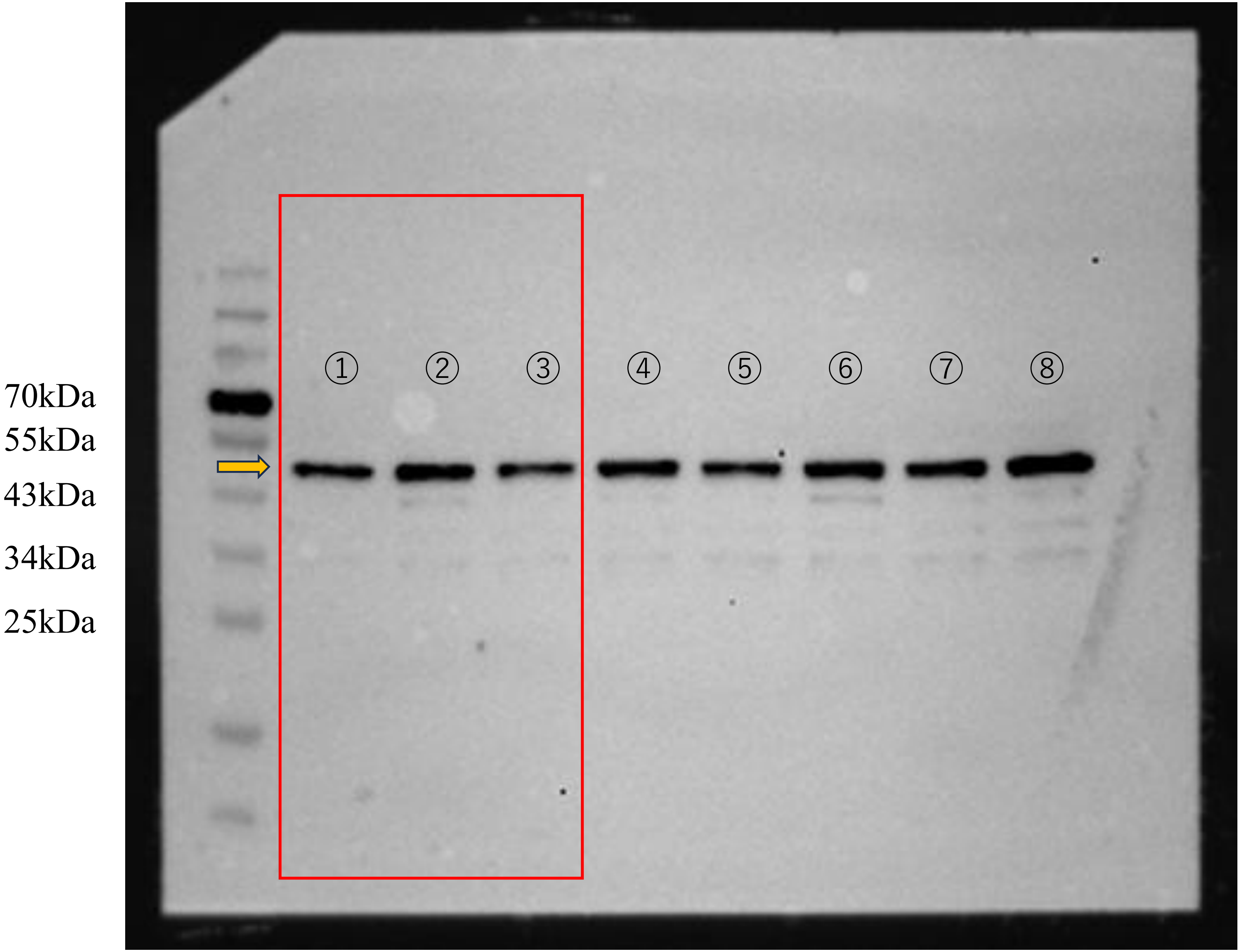

① Control (DMSO + Acetic acid)
② ET-1 (DMSO + ET-1)
③ CLZO + ET-1
④ Not used (Bosentan + ET-1)
⑤ Not used (DMSO + Acetic acid)
⑥ Not used (DMSO + ET-1)
⑦ Not used (CLZO + ET-1)
⑧ Not used (Bosentan + ET-1)

Arrow indicates the band used for quantification.

Full unedited blot for Figure 4c (β-actin)

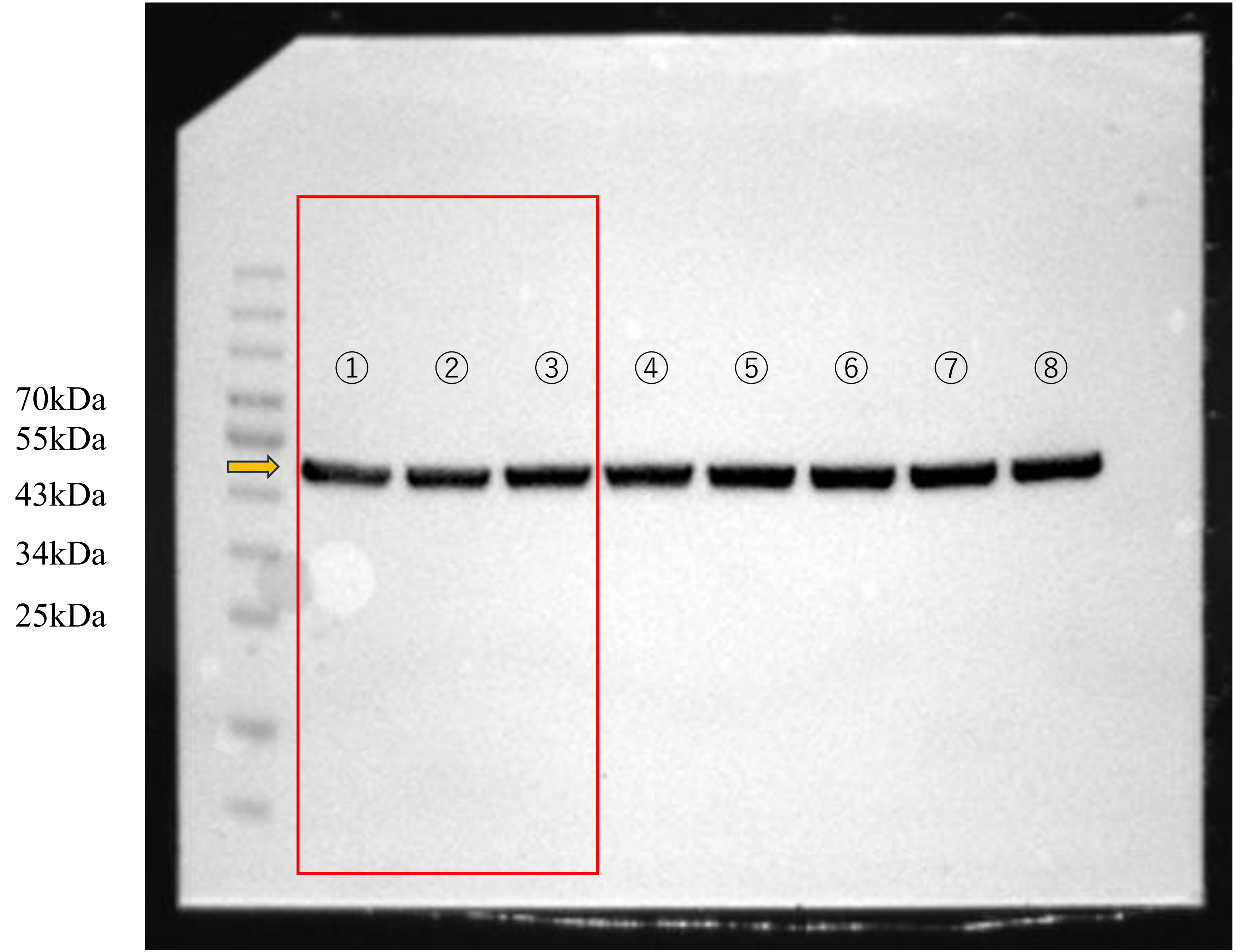

① Control (DMSO + Acetic acid)
② ET-1 (DMSO + ET-1)
③ CLZO + ET-1
④ Not used (Bosentan + ET-1)
⑤ Not used (DMSO + Acetic acid)
⑥ Not used (DMSO + ET-1)
⑦ Not used (CLZO + ET-1)
⑧ Not used (Bosentan + ET-1)

Arrow indicates the band used for quantification.
