# Supplementary material for: Clazosentan Attenuates Endothelin-1-Induced ETA Protein Upregulation and Contractile Sensitization in Brain Pericytes
Source: Pharmaceutics. 2026 Aug 9;18(8):980. doi: 10.3390/pharmaceutics18080980 (PMC13516358; doi:10.3390/pharmaceutics18080980)
Supplement: Supplementary file 1 [file pharmaceutics-18-00980-s001.zip › Supplementary Table S1.pdf]

Figure 1									
Figure/Panel	Outcome	Comparison / Model	Statistical test	Main effect / Contrast	Test statistic	Degrees of freedom	Exact p value	Multiple comparison method	Adjusted p value
Fig.1b (left panel)	Contraction index	Overall group effect across ET-1 concentrations (1, 10, 100, 1000 nM)	Kruskal–Wallis test	Omnibus	H(3) = 10.94	3	0.0016	Dunn's multiple-comparisons test	
	Contraction index	1 nM vs 10 nM	Dunn's multiple-comparisons test	Pairwise contrast				Dunn's multiple-comparisons test	0.4482
	Contraction index	1 nM vs 100 nM	Dunn's multiple-comparisons test	Pairwise contrast				Dunn's multiple-comparisons test	0.2255
	Contraction index	1 nM vs 1000 nM	Dunn's multiple-comparisons test	Pairwise contrast				Dunn's multiple-comparisons test	0.0065
	Contraction index	10 nM vs 100 nM	Dunn's multiple-comparisons test	Pairwise contrast				Dunn's multiple-comparisons test	>0.9999
	Contraction index	10 nM vs 1000 nM	Dunn's multiple-comparisons test	Pairwise contrast				Dunn's multiple-comparisons test	0.8249
	Contraction index	100 nM vs 1000 nM	Dunn's multiple-comparisons test	Pairwise contrast				Dunn's multiple-comparisons test	>0.9999
Fig.1b (right panel)	Dose-response trend	log10[ET-1 concentration] vs contraction index mean (n = 4 XY pairs)	Spearman's rank correlation	Association	ρ = 1.000		0.0833		
Fig.1c	Normalized cell index at 60 min (relative to control mean)	Overall group effect across ET-1 concentrations (1, 10, 100, 1000 nM)	Kruskal–Wallis test	Omnibus	H(3) = 4.456	3	0.2247	Dunn's multiple-comparisons test	
	Normalized cell index at 60 min (relative to control mean)	1 nM vs 10 nM	Dunn's multiple-comparisons test	Pairwise contrast				Dunn's multiple-comparisons test	0.8249
	Normalized cell index at 60 min (relative to control mean)	1 nM vs 100 nM	Dunn's multiple-comparisons test	Pairwise contrast				Dunn's multiple-comparisons test	0.3211
	Normalized cell index at 60 min (relative to control mean)	1 nM vs 1000 nM	Dunn's multiple-comparisons test	Pairwise contrast				Dunn's multiple-comparisons test	0.6139
	Normalized cell index at 60 min (relative to control mean)	10 nM vs 100 nM	Dunn's multiple-comparisons test	Pairwise contrast				Dunn's multiple-comparisons test	>0.9999
	Normalized cell index at 60 min (relative to control mean)	10 nM vs 1000 nM	Dunn's multiple-comparisons test	Pairwise contrast				Dunn's multiple-comparisons test	>0.9999
	Normalized cell index at 60 min (relative to control mean)	100 nM vs 1000 nM	Dunn's multiple-comparisons test	Pairwise contrast				Dunn's multiple-comparisons test	>0.9999

Figure 2	Outcome	Comparison / Model	Statistical test	Main effect / Contrast	Test statistic	Degrees of freedom	Exact p value	Multiple comparison method	Adjusted p value
Fig.2	Cell viability (% of control)	Across 8 ET-1 concentrations	Kruskal–Wallis test	Overall group effect	H = 11.89	7	0.1042		
	Cell viability (% of control)	Control vs 0.001 nM	Dunn’s multiple-comparisons test	Control vs 0.001 nM	Mean rank difference = 17.8			Dunn’s multiple-comparisons test	0.4498
	Cell viability (% of control)	Control vs 0.01 nM	Dunn’s multiple-comparisons test	Control vs 0.01 nM	Mean rank difference = 20.2			Dunn’s multiple-comparisons test	0.1762
	Cell viability (% of control)	Control vs 0.1 nM	Dunn’s multiple-comparisons test	Control vs 0.1 nM	Mean rank difference = 16.4			Dunn’s multiple-comparisons test	0.7433
	Cell viability (% of control)	Control vs 1 nM	Dunn’s multiple-comparisons test	Control vs 1 nM	Mean rank difference = 10.0			Dunn’s multiple-comparisons test	>0.9999
	Cell viability (% of control)	Control vs 10 nM	Dunn’s multiple-comparisons test	Control vs 10 nM	Mean rank difference = 20.4			Dunn’s multiple-comparisons test	0.1623
	Cell viability (% of control)	Control vs 100 nM	Dunn’s multiple-comparisons test	Control vs 100 nM	Mean rank difference = 13.0			Dunn’s multiple-comparisons test	>0.9999
	Cell viability (% of control)	Control vs 1000 nM	Dunn’s multiple-comparisons test	Control vs 1000 nM	Mean rank difference = 10.2			Dunn’s multiple-comparisons test	>0.9999
	Cell viability (% of control)	0.001 vs 0.01 nM	Dunn’s multiple-comparisons test	0.001 vs 0.01 nM	Mean rank difference = 2.4			Dunn’s multiple-comparisons test	>0.9999
	Cell viability (% of control)	0.001 vs 0.1 nM	Dunn’s multiple-comparisons test	0.001 vs 0.1 nM	Mean rank difference = -1.4			Dunn’s multiple-comparisons test	>0.9999
	Cell viability (% of control)	0.001 vs 1 nM	Dunn’s multiple-comparisons test	0.001 vs 1 nM	Mean rank difference = -7.8			Dunn’s multiple-comparisons test	>0.9999
	Cell viability (% of control)	0.001 vs 10 nM	Dunn’s multiple-comparisons test	0.001 vs 10 nM	Mean rank difference = 2.6			Dunn’s multiple-comparisons test	>0.9999
	Cell viability (% of control)	0.001 vs 100 nM	Dunn’s multiple-comparisons test	0.001 vs 100 nM	Mean rank difference = -4.8			Dunn’s multiple-comparisons test	>0.9999
	Cell viability (% of control)	0.001 vs 1000 nM	Dunn’s multiple-comparisons test	0.001 vs 1000 nM	Mean rank difference = -7.6			Dunn’s multiple-comparisons test	>0.9999
	Cell viability (% of control)	0.01 vs 0.1 nM	Dunn’s multiple-comparisons test	0.01 vs 0.1 nM	Mean rank difference = -3.8			Dunn’s multiple-comparisons test	>0.9999
	Cell viability (% of control)	0.01 vs 1 nM	Dunn’s multiple-comparisons test	0.01 vs 1 nM	Mean rank difference = -10.2			Dunn’s multiple-comparisons test	>0.9999
	Cell viability (% of control)	0.01 vs 10 nM	Dunn’s multiple-comparisons test	0.01 vs 10 nM	Mean rank difference = 0.2			Dunn’s multiple-comparisons test	>0.9999
	Cell viability (% of control)	0.01 vs 100 nM	Dunn’s multiple-comparisons test	0.01 vs 100 nM	Mean rank difference = -7.2			Dunn’s multiple-comparisons test	>0.9999
	Cell viability (% of control)	0.01 vs 1000 nM	Dunn’s multiple-comparisons test	0.01 vs 1000 nM	Mean rank difference = -10.0			Dunn’s multiple-comparisons test	>0.9999
	Cell viability (% of control)	0.1 vs 1 nM	Dunn’s multiple-comparisons test	0.1 vs 1 nM	Mean rank difference = -6.4			Dunn’s multiple-comparisons test	>0.9999
	Cell viability (% of control)	0.1 vs 10 nM	Dunn’s multiple-comparisons test	0.1 vs 10 nM	Mean rank difference = 4.0			Dunn’s multiple-comparisons test	>0.9999

Figure 2	Outcome	Comparison / Model	Statistical test	Main effect / Contrast	Test statistic	Degrees of freedom	Exact p value	Multiple comparison method	Adjusted p value
	Cell viability (% of control)	0.1 vs 100 nM	Dunn's multiple-comparisons test	0.1 vs 100 nM	Mean rank difference = -3.4			Dunn's multiple-comparisons test	>0.9999
	Cell viability (% of control)	0.1 vs 1000 nM	Dunn's multiple-comparisons test	0.1 vs 1000 nM	Mean rank difference = -6.2			Dunn's multiple-comparisons test	>0.9999
	Cell viability (% of control)	1 vs 10 nM	Dunn's multiple-comparisons test	1 vs 10 nM	Mean rank difference = 10.4			Dunn's multiple-comparisons test	>0.9999
	Cell viability (% of control)	1 vs 100 nM	Dunn's multiple-comparisons test	1 vs 100 nM	Mean rank difference = 3.0			Dunn's multiple-comparisons test	>0.9999
	Cell viability (% of control)	1 vs 1000 nM	Dunn's multiple-comparisons test	1 vs 1000 nM	Mean rank difference = 0.2			Dunn's multiple-comparisons test	>0.9999
	Cell viability (% of control)	10 vs 100 nM	Dunn's multiple-comparisons test	10 vs 100 nM	Mean rank difference = -7.4			Dunn's multiple-comparisons test	>0.9999
	Cell viability (% of control)	10 vs 1000 nM	Dunn's multiple-comparisons test	10 vs 1000 nM	Mean rank difference = -10.2			Dunn's multiple-comparisons test	>0.9999
	Cell viability (% of control)	100 vs 1000 nM	Dunn's multiple-comparisons test	100 vs 1000 nM	Mean rank difference = -2.8			Dunn's multiple-comparisons test	>0.9999

Figure 4	Outcome	Comparison / Model	Statistical test	Main effect / Contrast	Test statistic	Degrees of freedom	Exact p value	Multiple comparison method	Adjusted p value
Fig.4a	Time-course comparison	Overall model	Kruskal-Wallis test	Omnibus	H = 9.291	3	0.0101		
	Time-course comparison	Control vs. 24	Dunn's multiple-comparisons test	Pairwise contrast				Dunn's	0.2603
	Time-course comparison	Control vs. 48	Dunn's multiple-comparisons test	Pairwise contrast				Dunn's	0.0268
	Time-course comparison	Control vs. 72	Dunn's multiple-comparisons test	Pairwise contrast				Dunn's	0.1223
Fig.4b (left panel)	Dose-response comparison	Overall model	Kruskal-Wallis test	Omnibus	H = 10.9	6	0.0914		
	Dose-response comparison	Control vs. 0.01	Dunn's multiple-comparisons test	Pairwise contrast				Dunn's	>0.9999
	Dose-response comparison	Control vs. 0.1	Dunn's multiple-comparisons test	Pairwise contrast				Dunn's	>0.9999
	Dose-response comparison	Control vs. 1	Dunn's multiple-comparisons test	Pairwise contrast				Dunn's	0.8635
	Dose-response comparison	Control vs. 10	Dunn's multiple-comparisons test	Pairwise contrast				Dunn's	0.7353
	Dose-response comparison	Control vs. 100	Dunn's multiple-comparisons test	Pairwise contrast				Dunn's	>0.9999
	Dose-response comparison	Control vs. 1000	Dunn's multiple-comparisons test	Pairwise contrast				Dunn's	0.3718
Fig.4b (right panel)	Dose-response trend	log10[ET-1 concentration] vs ETA protein abundance mean (n = 6 XY pairs)	Spearman's rank correlation	Association	$\rho = 1.000$		0.0028		
Fig.4c	Group comparison	Overall model	Kruskal-Wallis test	Omnibus	H = 20.42	2	<0.0001		
	Group comparison	Control vs. ET-1	Dunn's multiple-comparisons test	Pairwise contrast				Dunn's	<0.0001
	Group comparison	Control vs. ET-1 + CLZO	Dunn's multiple-comparisons test	Pairwise contrast				Dunn's	0.1970
	Group comparison	ET-1 vs. ET-1 + CLZO	Dunn's multiple-comparisons test	Pairwise contrast				Dunn's	0.0238

Figure 5	Outcome	Comparison / Model	Statistical test	Main effect / Contrast	Test statistic	Degrees of freedom	Exact p value	Multiple comparison method	Adjusted p value
Fig.5b	Relative contraction index	Overall model	Brown–Forsythe and Welch ANOVA	Group effect	F* = 11.99; W = 12.65	(2, 14.08); (2, 19.61)	0.0009; 0.0003		
	Relative contraction index	Control vs ET-1	Post hoc contrast	Mean difference	t = 3.826	13.14		Dunnett's T3	0.0062
	Relative contraction index	Control vs ET-1 + CLZO	Post hoc contrast	Mean difference	t = 3.495	16.77		Dunnett's T3	0.0082
	Relative contraction index	ET-1 vs ET-1 + CLZO	Post hoc contrast	Mean difference	t = 3.069	13.94		Dunnett's T3	0.0241
Fig.5c	Normalized cell index at 60 min	Overall model	Ordinary one-way ANOVA	Treatment effect	F = 11.05	(2, 39)	0.0002		
	Normalized cell index at 60 min	Control vs ET-1	Post hoc contrast	Mean difference	q = 6.583	39		Tukey	0.0001
	Normalized cell index at 60 min	Control vs CLZO + ET-1	Post hoc contrast	Mean difference	q = 2.477	39		Tukey	0.1994
	Normalized cell index at 60 min	ET-1 vs CLZO + ET-1	Post hoc contrast	Mean difference	q = 4.106	39		Tukey	0.0163

Figure 6									
Figure / Panel	Outcome	Comparison / Model	Statistical test	Main effect / Contrast	Test statistic	Degrees of freedom	Exact p value	Multiple comparison method	Adjusted p value
Fig.6a	TEER (Ω·cm²)	Treatment × Time model	Two-way repeated-measures ANOVA	Interaction	F=0.8737	(1.387, 8.325)	0.4133		
	TEER (Ω·cm²)	Treatment × Time model	Two-way repeated-measures ANOVA	Time main effect	F=31.33	(1.387, 8.325)	0.0002		
	TEER (Ω·cm²)	Treatment × Time model	Two-way repeated-measures ANOVA	Treatment main effect	F=0.1201	(1, 6)	0.7408		
	TEER (Ω·cm²)	Control vs CLZO at 0 h	Post hoc contrast	Between-group contrast at 0 h	t=0.3486	5.874		Šídák	0.7396
	TEER (Ω·cm²)	Control vs CLZO at 24 h	Post hoc contrast	Between-group contrast at 24 h	t=0.4772	5.804		Šídák	0.6506
	TEER (Ω·cm²)	Control vs CLZO at 48 h	Post hoc contrast	Between-group contrast at 48 h	t=0.1869	5.997		Šídák	0.8579
Fig.6a	Normalized TEER (relative to control)	Treatment × Time model	Two-way repeated-measures ANOVA	Interaction	F=1.408	(1.760, 10.56)	0.2836		
	Normalized TEER (relative to control)	Treatment × Time model	Two-way repeated-measures ANOVA	Time main effect	F=1.408	(1.760, 10.56)	0.2836		
	Normalized TEER (relative to control)	Treatment × Time model	Two-way repeated-measures ANOVA	Treatment main effect	F=0.0001894	(1, 6)	0.9895		
	Normalized TEER (relative to control)	Control vs CLZO at 0 h	Post hoc contrast	Between-group contrast at 0 h	t=1.442×10^-7	5.955		Šídák	>0.9999
	Normalized TEER (relative to control)	Control vs CLZO at 24 h	Post hoc contrast	Between-group contrast at 24 h	t=0.1367	5.908		Šídák	0.8958
	Normalized TEER (relative to control)	Control vs CLZO at 48 h	Post hoc contrast	Between-group contrast at 48 h	t=0.1884	5.990		Šídák	0.8568

Figure 6									
Fig.6b	TEER (Ω·cm²)	Treatment × Time model	Two-way repeated-measures ANOVA	Interaction	F=1.291	(2, 18)	0.2992		
	TEER (Ω·cm²)	Treatment × Time model	Two-way repeated-measures ANOVA	Time main effect	F=25.35	(2, 18)	<0.0001		
	TEER (Ω·cm²)	Treatment × Time model	Two-way repeated-measures ANOVA	Treatment main effect	F=0.3336	(1, 18)	0.5707		
	TEER (Ω·cm²)	Control vs CLZO at 0 h	Post hoc contrast	Between-group contrast at 0 h	t=0.3741	18		Šídák	0.7127
	TEER (Ω·cm²)	Control vs CLZO at 24 h	Post hoc contrast	Between-group contrast at 24 h	t=1.449	18		Šídák	0.1646
	TEER (Ω·cm²)	Control vs CLZO at 48 h	Post hoc contrast	Between-group contrast at 48 h	t=0.8226	18		Šídák	0.4215
Fig.6b	Normalized TEER (relative to control)	Treatment × Time model	Two-way repeated-measures ANOVA	Interaction	F=3.036	(1.128, 6.768)	0.1251		
	Normalized TEER (relative to control)	Treatment × Time model	Two-way repeated-measures ANOVA	Time main effect	F=3.036	(1.128, 6.768)	0.1251		
	Normalized TEER (relative to control)	Treatment × Time model	Two-way repeated-measures ANOVA	Treatment main effect	F=0.01418	(1, 6)	0.9091		
	Normalized TEER (relative to control)	Control vs CLZO at 0 h	Post hoc contrast	Between-group contrast at 0 h	t=9.594×10^-6	5.819		Šídák	>0.9999
	Normalized TEER (relative to control)	Control vs CLZO at 24 h	Post hoc contrast	Between-group contrast at 24 h	t=1.144	4.836		Šídák	0.3061
	Normalized TEER (relative to control)	Control vs CLZO at 48 h	Post hoc contrast	Between-group contrast at 48 h	t=1.25	5.269		Šídák	0.2640
Fig.6c	TEER (Ω·cm²)	Treatment × Time model	Two-way repeated-measures ANOVA	Interaction	F=0.08708	(1.165, 6.990)	0.8132		
	TEER (Ω·cm²)	Treatment × Time model	Two-way repeated-measures ANOVA	Time main effect	F=57.21	(1.165, 6.990)	0.0001		
	TEER (Ω·cm²)	Treatment × Time model	Two-way repeated-measures ANOVA	Treatment main effect	F=0.5029	(1, 6)	0.5049		
	TEER (Ω·cm²)	Control vs ET-1 at 0 h	Post hoc contrast	Between-group contrast at 0 h	t=0.8899	5.667		Šídák	0.4097
	TEER (Ω·cm²)	Control vs ET-1 at 24 h	Post hoc contrast	Between-group contrast at 24 h	t=0.6831	5.534		Šídák	0.5221
	TEER (Ω·cm²)	Control vs ET-1 at 48 h	Post hoc contrast	Between-group contrast at 48 h	t=0.3709	3.393		Šídák	0.7327
Fig.6c	Normalized TEER (relative to control)	Treatment × Time model	Two-way repeated-measures ANOVA	Interaction	F=0.06522	(1.136, 6.813)	0.8357		
	Normalized TEER (relative to control)	Treatment × Time model	Two-way repeated-measures ANOVA	Time main effect	F=0.06522	(1.136, 6.813)	0.8357		
	Normalized TEER (relative to control)	Treatment × Time model	Two-way repeated-measures ANOVA	Treatment main effect	F=0.03421	(1, 6)	0.8593		
	Normalized TEER (relative to control)	Control vs ET-1 at 0 h	Post hoc contrast	Between-group contrast at 0 h	t=3.111×10^-6	5.734		Šídák	>0.9999

Figure 6									
	Normalized TEER (relative to control)	Control vs ET-1 at 24 h	Post hoc contrast	Between-group contrast at 24 h	t=0.1920	5.609		Šídák	0.8545
	Normalized TEER (relative to control)	Control vs ET-1 at 48 h	Post hoc contrast	Between-group contrast at 48 h	t=0.2553	3.371		Šídák	0.8133

Supplementary Table S1. Full statistical results for Figures 1, 2, 4, 5, and 6.

Statistical results are presented as omnibus test results and figure-relevant post hoc comparisons. Main effects, interaction effects, degrees of freedom, test statistics, and exact p values are reported where applicable. Adjusted p values are provided for post hoc multiple comparisons.
